# Targeted Mitochondrial ECSIT Overexpression Attenuates MASH by Increasing OTUD3 Expression

**DOI:** 10.1002/advs.202518974

**Published:** 2026-02-04

**Authors:** Yuqing Jiang, Tingting Tong, Pengxi Shi, Xiaofan Chen, Chenhao Wang, Qingyuan Weng, Sihan Chen, Linli Que, Qi Chen, Yuehua Li, Qiang Zhu, Jiantao Li

**Affiliations:** ^1^ Key Laboratory of Targeted Intervention of Cardiovascular Disease Collaborative Innovation Center for Cardiovascular Disease Translational Medicine School of Basic Medical Science Nanjing Medical University Nanjing Jiangsu China; ^2^ The First Clinical Medical College Nanjing Medical University Nanjing Jiangsu China; ^3^ Department of Organ Transplantation Eastern Hepatobiliary Surgery Hospital Naval Medical University Shanghai China

**Keywords:** ECSIT, MASH, OTUD3, ox‐mtDNA, SIRT3

## Abstract

Mitochondrial dysfunction plays a key role in the pathogenesis of metabolic dysfunction‐associated steatohepatitis (MASH). As is known to play a key role in mitochondria, ECSIT, in relation to oxidized mitochondrial DNA is still unclear. This study examines mitochondrial ECSIT expression in MASH mouse models. Mitochondria‐targeted ECSIT transgenic (ECSIT^MTG^) mice and wild‐type (WT) controls are fed a high‐fat, high‐cholesterol (HFHC) diet for 16 weeks or a methionine‐ and choline‐deficient (MCD) diet for 8 weeks. Results demonstrate that mitochondrial ECSIT overexpression alleviates diet‐induced MASH phenotypes. Mechanistically, we demonstrate that mitochondrial ECSIT promotes the localization of the deubiquitinase OTUD3 to mitochondria. OTUD3 then stabilizes SIRT3 via deubiquitination, thereby inhibiting mtDNA oxidation and alleviating steatosis‐induced metabolic disorders. Overall, these findings indicate that mitochondrial ECSIT protects against MASH progression by stabilizing SIRT3, suggesting its potential as a therapeutic target.

## Graphical abstract

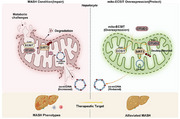

## 1 Introduction

As a hepatic manifestation of metabolic syndrome, metabolic dysfunction‐associated steatotic liver disease (MASLD) is affecting approximately one billion individuals worldwide [1]. Frequently coexisting with and exacerbating type 2 diabetes and cardiovascular disease, MASLD is intrinsically linked to insulin resistance, visceral adiposity, and atherogenic dyslipidemia [2]. MASLD can progress to metabolic dysfunction‐associated steatohepatitis (MASH). This advanced stage is characterized by persistent hepatic inflammation and fibrosis. Notably, the inflammatory response is a central driver of hepatocyte injury. This progression significantly increases the risk of severe complications, including cirrhosis and hepatocellular carcinoma (HCC) [3]. However, among the therapies approved, just few directly target MASH‐related inflammation, thus underscoring the urgence for mechanistic explorations to brew novel treatments [4].

Liver function is closely related with the status of mitochondria [5, 6]. In MASH, mitochondrial failure drives hepatic inflammation beyond metabolic dysregulation [7]. In particular, mitochondrial DNA (mtDNA) displays a histone‐free structure and a proximity to reactive oxygen species (ROS)‐generating sites, thus its damage under metabolic stressors elicits inflammatory processes, manifesting as respiratory chain collapse, ROS overproduction, and lipid accumulation. Moreover, cytosolic mtDNA fragments activate pattern recognition receptors (PRRs), thereby triggering potent innate immunity that maintains the pro‐inflammatory milieu in MASH. As the predominant mitochondrial Sirtuins, SIRT3 (Sirtuin 3) governs the acetylation of over 60% of mitochondrial proteins, including key enzymes in the tricarboxylic acid cycle, fatty acid β‐oxidation machinery, and antioxidant defense systems [8]. The seminal work by Zhou et al. suggests that SIRT3 mediates the deacetylation of Superoxide Dismutase 2 (SOD2) to enhance its enzymatic activity essential for quenching mitochondrial superoxide and safeguarding mtDNA integrity in experimental silicosis [9]. Moreover, SIRT3 contributes to mitochondrial genome maintenance by directly regulating base excision repair proteins, such as Neendonuclease I‐Like 1, Neendonuclease I‐Like 2, and Ligase 3 [10]. Critically, hepatic SIRT3 downregulation is consistently observed in the progression of either MASLD or MASH, yet the underpinning mechanisms remain elusive [11].

Accumulating evidence has established the critical roles of mitochondrial protein ubiquitination and deubiquitination in maintaining mitochondrial integrity and cellular homeostasis [12, 13, 14]. However, the potential involvement of SIRT3 ubiquitination and deubiquitination in MASH pathogenesis remains unknown. Ovarian Tumor Domain Containing Protein 3 (OTUD3), a catalytically active member of the ovarian tumor domain deubiquitinase superfamily, distributes ubiquitously in the tissues of mammals and functions as a metabolic rheostat responsive to nutritional status [15]. Emerging evidence putatively indicates that OTUD3 may locate into the mitochondria, thereby modifying current understandings about the distribution of deubiquitinating enzymes [16]. Nevertheless, the mechanism underlying this intramitochondrial localization in the absence of a canonical mitochondrial targeting sequence (MTS), as well as the mitochondrial molecular substrates of OTUD3, remain unresolved.

Phylogenetically conserved across metazoans, evolutionary conserved signaling intermediate in Toll pathways (ECSIT) functions as a pivotal adaptor protein in mitochondrial bioenergetics [17]. Subcellular proteomic mapping has established its dual compartmentalization. Mitochondrial‐localized ECSIT plays a dual role in regulating mitochondrial redox signaling: it promotes Complex I biogenesis by assembling with NADH dehydrogenase 1 alpha factor 1/ Acyl‐CoA dehydrogenase 9, and enhances mitochondrial reactive oxygen species (mROS) production by acting on TRAF6‐mediated ubiquitination [18, 19]. Specifically, ECSIT interacts with signal transducer and activator of transcription 3 (STAT3) to guard mitochondrial function during pathological cardiac hypertrophy [20]. In Alzheimer's disease, ECSIT protects against neuropathy by preserving mitochondrial function [21]. Despite these findings, the pathophysiological implications of ECSIT‐mediated mitochondrial quality control remain uncharted in hepatocyte lipotoxicity and MASH‐associated mitochondrial dysregulation.

This study was the first ever to demonstrate a reduction in mitochondrial ECSIT protein levels in the livers of mice with MASH induced by specific diets. Mitochondrial ECSIT upregulates the intramitochondrial expression of deubiquitinase OTUD3 and ensures SIRT3 protein stability to alleviate oxidized mitochondrial DNA (ox‐mtDNA) and counter the progression of MASH.

## 2 Results

### 2.1 Mitochondrial ECSIT Protein Expression is Reduced in MASH Models

**FIGURE 1:**
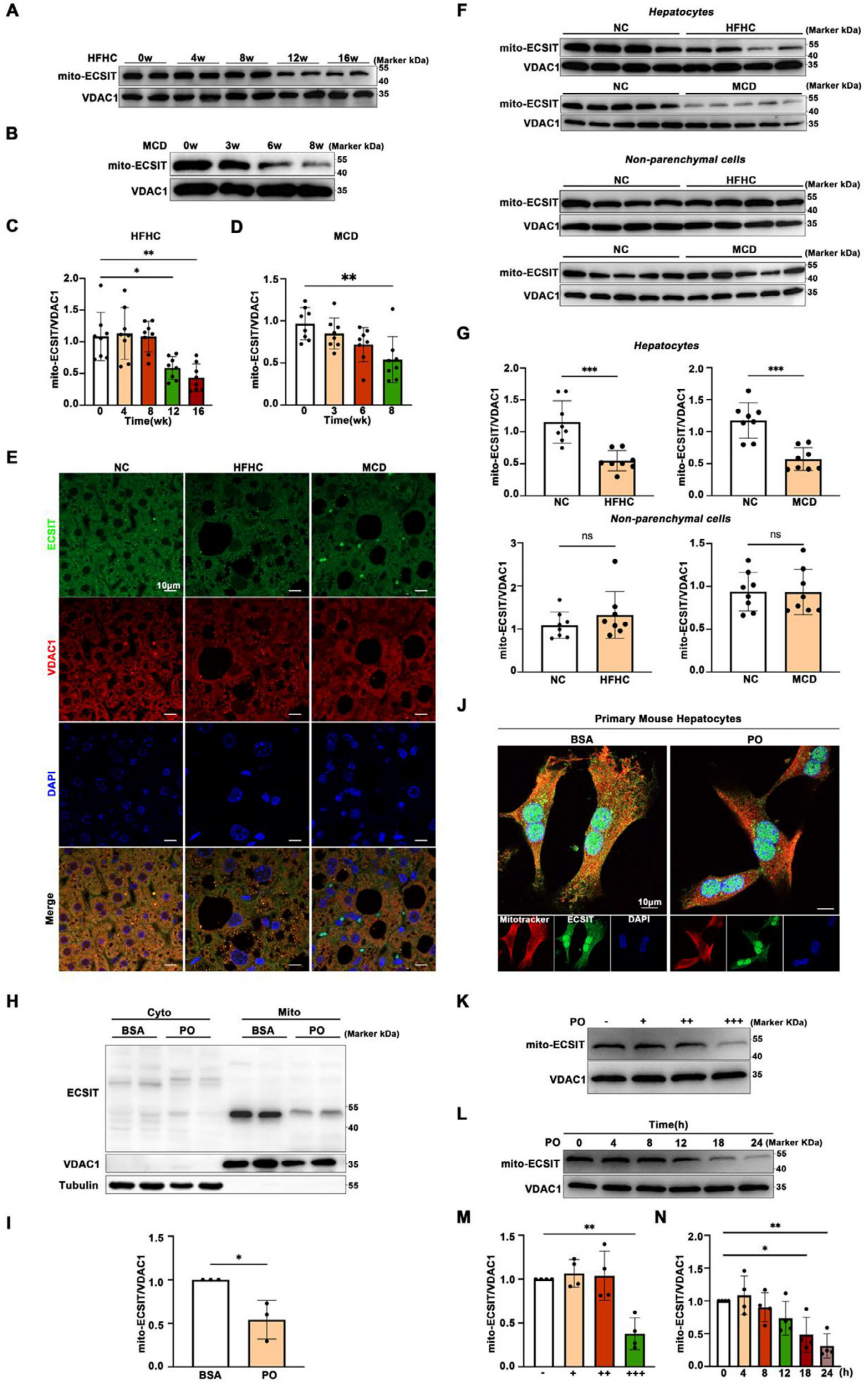
Mitochondrial ECSIT is downregulated in MASH. A) Mice were fed an HFHC diet for the indicated durations (0, 4, 8, 12, and 16 weeks). Hepatic expression of mito‐ECSIT was assessed by western blot analysis. *n = 8*. B) Mice were fed an MCD diet for the indicated durations (0, 3, 6, and 8 weeks). Hepatic expression of mito‐ECSIT was assessed by western blot analysis. *n = 8*. C) Statistical analysis of A. D) Statistical analysis of B. E) Representative immunofluorescence images of mouse liver sections from NC, HFHC, and MCD diet‐fed mice. Sections were co‐stained with antibodies against VDAC1 and ECSIT to assess their co‐localization. *n = 8*. Scale bar: 10 µm. F) Western blot analysis of mitochondrial ECSIT expression in primary hepatocytes and non‐parenchymal cells isolated from diet‐induced MASH models and control mice. *n = 8*. G) Statistical analysis of F. H) Representative Western blotting and statistical analysis of mitochondrial ECSIT in the primary mouse hepatocytes treated with BSA or PO for 24 h. *n = 3*. I) Statistical analysis of H. J) Representative immunofluorescence images of mitochondria and ECSIT in primary mouse hepatocytes treated with BSA or PO for 24 h. *n = 4*. Scale bar: 100 µm. K) Primary mouse hepatocytes were treated with PO (PA:OA = 3:5 ratio) at the indicated concentrations (PA: 0, 0.1, 0.2, and 0.3mm) for 24 h. Mito‐ECSIT protein levels were assessed by western blotting. *n = 4*. L) Primary mouse hepatocytes were treated with PO (0.3 mM PA and 0.5mM OA) and harvested at the indicated time points. Protein levels of the mito‐ECSIT were analyzed by Western blot. *n = 4*. M) Statistical analysis of K. N) Statistical analysis of L. All data were shown as the mean ± SD. Statistical analyses were performed by one‐way ANOVA followed by Tukey's tests for multiple comparisons and two‐tailed Student's *t*‐test between two groups. ^*^ *P* < 0.05, ^**^ *P* < 0.01, ^***^ *P* < 0.001, ns indicates not significant.

To delineate the alterations in mitochondrial ECSIT expression during metabolic stress, we employed both in vivo dietary models and in vitro cellular experiments. Western blot analysis of isolated liver mitochondria from C57BL/6J mice revealed distinct temporal patterns of ECSIT downregulation depending on the dietary challenge. In mice fed a high‐fat, high‐cholesterol (HFHC) diet, mitochondrial ECSIT protein levels remained comparable to controls in the early phases but exhibited a significant decrease at 12 weeks, which was further pronounced after 16 weeks of feeding (Figure 1A,C). Notably, in the methionine‐choline deficient (MCD) diet model, a downward trend in mitochondrial ECSIT expression was detectable as early as 3 weeks, progressing to a statistically significant reduction by 8 weeks (Figure 1B,D). Immunofluorescence staining of liver sections confirmed these findings, demonstrating a marked decrease in mitochondrial‐localized ECSIT signal in both HFHC and MCD diet‐induced mouse models of MASH compared to their respective control groups (Figure 1E). Furthermore, the mitochondrial ECSIT protein level remained unchanged in the non‐parenchymal cell fraction but was specifically altered in hepatocytes isolated from MASH mice (Figure 1F,G).

We further investigated this phenomenon in vitro using primary hepatocytes. Stimulation with palmitic acid and oleic acid (PO) to mimic lipotoxic stress led to a significant downregulation of mitochondrial ECSIT protein after 24 h, as confirmed by both Western blot and immunofluorescence analysis (Figure 1H–J). To determine the condition dependency of this response, we treated primary hepatocytes with varying concentrations or different durations of PO. The results indicated that the reduction in mitochondrial ECSIT expression was not universal but required specific thresholds; a significant decrease was observed only upon exposure to a high concentration of PO or after a prolonged stimulation period, whereas lower concentrations or shorter exposures did not elicit a significant change (Figure 1K–N). Collectively, our findings demonstrate that downregulation of mitochondrial ECSIT is a characteristic feature in the livers of MASH mice.

To investigate the function of mitochondrial ECSIT in MASH pathogenesis, we constructed an adenoviral vector for targeted mitochondrial expression. This vector expresses full‐length ECSIT fused to an N‐terminal mitochondrial targeting sequence (MTS) derived from ornithine transcarbamylase (OTC), designated Adv‐otcl‐ECSIT‐Flag, with a corresponding control vector (Adv‐Ctrl). The OTC presequence is a canonical and potent signal that ensures efficient import of the fusion protein into the mitochondrial matrix, thereby achieving robust mitochondrial localization. Western blot analysis revealed a band at approximately 55 kDa, corresponding to mitochondrial ECSIT, which confirmed successful transduction and mitochondrial targeting (Figure S1A). Oil Red O staining showed that targeting mitochondria ECSIT overexpression reduced lipid accumulation in PO‐challenged hepatocytes, compared to controls (Figure S1B). Furthermore, quantitative PCR analysis revealed that ECSIT overexpression significantly attenuated PO‐induced upregulation of proinflammatory cytokines, including *Tnf*, *Il‐6*, *Il‐1b*, and *Cxcl2* (Figure S1C). These findings collectively demonstrate that mitochondrial ECSIT exerts protective effects against hepatocyte lipotoxicity by concurrently ameliorating both lipid overload and inflammatory responses.

### 2.2 Targeted Mitochondrial ECSIT Overexpression Curbs HFHC‐Induced MASH Progression in Mice

**FIGURE 2:**
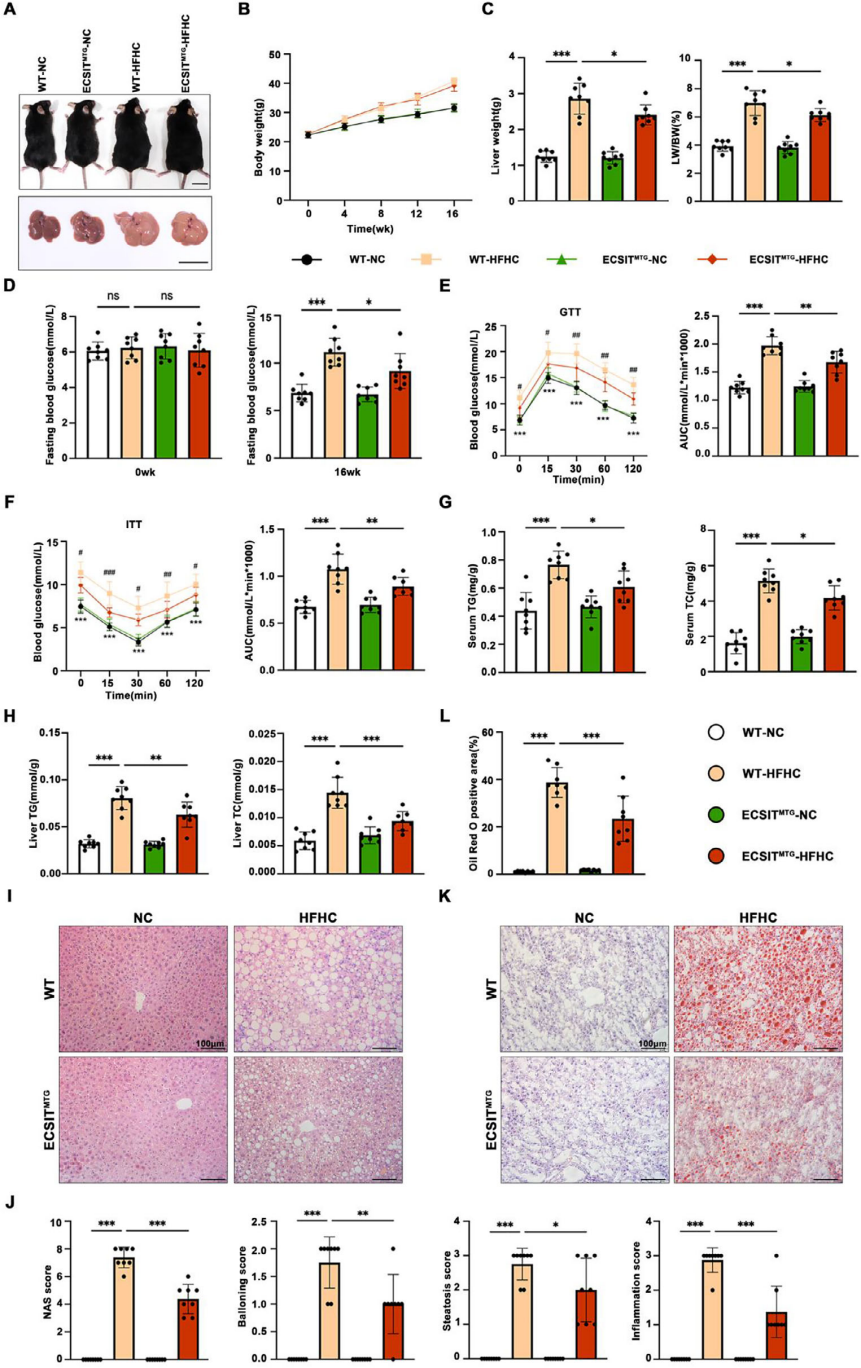
Mitochondrial‐targeted overexpression of ECSIT alleviates hepatic steatosis and improves glucose metabolism in HFHC diet‐induced MASH. WT and ECSIT^MTG^ mice were fed with NC or HFHC diets for 16 weeks. A) Representative body images and liver images in the indicated groups. Scale bar: 2 cm. B‐H) Records for the body weight (B), liver weight and the ratio of liver weight/body weight (%) (C), fasting blood glucose levels (D), glucose tolerance test (GTT) (E), insulin tolerance test (ITT) (F), serum TG and TC levels (G), liver TG and TC levels (H) in the indicated groups. *n = 8*. I,J) Representative pictures of H&E staining (I) and histological NAS score (J) in the liver sections of mice in the indicated groups. *n = 8*. Scale bar: 100 µm. K, L) Representative pictures of Oil Red O staining (K) and statistical analysis (L) showing the lipid accumulation in the liver sections of mice in the indicated groups. *n = 8*. Scale bar: 100 µm. All data were shown as the mean ± SD. Statistical analyses were performed by two‐way ANOVA followed by Tukey's tests for body weight and one‐way ANOVA followed by Tukey's tests for multiple comparisons. ^*^ *P* < 0.05, ^**^ *P *< 0.01, ^***^ *P* < 0.001, ns indicates not significant.

To address the function of mitochondrial ECSIT in MASH in vivo, we generated mitochondrial ECSIT transgenic mice (ECSIT^MTG^, Figure S2A–C), controlled with wildtype mice (WT). The mice were subjected to either a normal chow (NC) or HFHC diet for 16 weeks. Western blotting demonstrated that mitochondrial ECSIT expression was successfully increased in the livers of ECSIT^MTG^ mice, compared to WT mice (Figure S2C). After 16 weeks of HFHC feeding, the body weight of WT or ECSIT^MTG^ mice was significantly increased compared with their control group (Figure 2A,B). Compared to the WT mice, ECSIT^MTG^ mice exhibited a lower liver weight and a lower liver‐to body weight ratio, but no significant difference in the bodyweight under HFHC conditions (Figure 2A–C). ECSIT^MTG^ mice fed with HFHC diet showed a lower fasting serum glucose concentration (Figure 2D) and stronger glucose and insulin tolerances than WT mice, as assessed by glucose tolerance tests (GTT) and insulin tolerance tests (ITT) (Figure 2E,F). HFHC diet‐induced hepatic steatosis, as shown by the triglyceride (TG) and cholesterol (TC) levels in the serum and liver, was also markedly attenuated in ECSIT^MTG^ mice (Figure 2G,H). Histological analysis using H&E staining, along with the NAFLD activity score (NAS), indicated that mitochondria targeted ECSIT overexpression significantly reduced hepatic steatosis (Figure 2I,J). Oil Red O staining showed the same changes (Figure 2K,L). These results indicate that overexpression of mitochondrial ECSIT alleviates hepatic steatosis during the progression of MASH.

**FIGURE 3:**
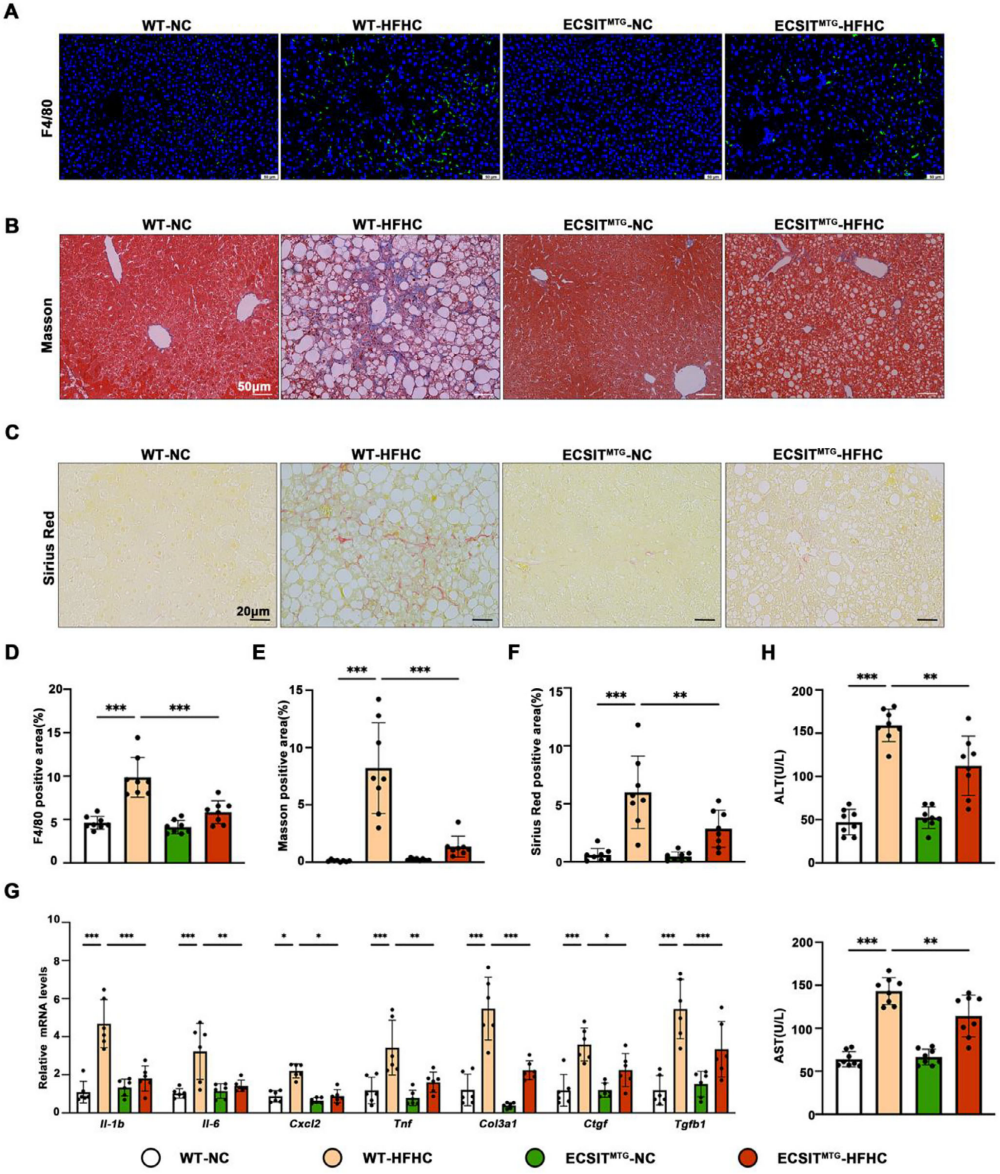
Mitochondrial ECSIT overexpression attenuates hepatic inflammation, fibrosis, and injury in HFHC diet‐induced MASH. WT and ECSIT^MTG^ mice were fed with NC or HFHC diets for 16 weeks. A) Representative pictures of immunofluorescence staining of F4/80 (green) in the liver sections of mice in the indicated groups (Nuclei, blue). *n = 8*. Scale bar: 50 µm. B) Representative pictures of Masson staining indicated liver fibrosis changes in the liver sections of mice in the indicated groups. *n = 8*. Scale bar: 50 µm. C) Representative pictures of Sirius Red staining indicated liver fibrosis changes in the liver sections of mice in the indicated groups. *n = 8*. Scale bar: 20 µm. D–F) Statistical analysis of F4/80 positive area (D), Masson positive area (E), Sirius Red positive area (F). G) qPCR was performed to analyze mRNA expression of proinflammation‐related genes (*Tnf*, *Il‐6*, *Il‐1b*, and *Cxcl2*) and fibrosis‐related genes (*Col3a1*, *Ctgf*, and *Tgfb1*). *n = 6*. H) Serum ALT and AST concentrations of mice in the indicated groups. *n = 8*. All data were shown as the mean ± SD. Statistical analyses were performed by two‐way ANOVA followed by Tukey's tests for body weight and one‐way ANOVA followed by Tukey's tests for multiple comparisons. ^*^ *P* < 0.05, ^**^ *P *< 0.01, ^***^ *P* < 0.001.

HFHC‐fed ECSIT^MTG^ mice also exhibited milder inflammatory responses than WT mice, as evidenced by the decreased infiltration of F4/80 positive inflammatory cells (Figure 3A,D) and lower mRNA expression levels of cytokines (*Tnf, Il‐6, Il‐1b*, and *Cxcl2*; Figure 3G) in the liver. The deposition of collagens (Figure 3B,C,E,F) and the expression of fibrogenic genes (*Col3a1*, *Ctgf*, and *Tgfb1*; Figure 3G) were also lower in the livers of HFHC‐fed ECSIT^MTG^ mice than WT mice. Furthermore, serum ALT and AST levels were significantly lower in ECSIT^MTG^ mice following HFHC diet consumption (Figure 3H). Taken all together, mitochondria targeted ECSIT overexpression curbs the progression of inflammation and fibrosis during MASH.

### 2.3 Targeted Mitochondrial ECSIT Overexpression Impedes MCD‐Induced MASH Progression in Transgenic Mice

**FIGURE 4:**
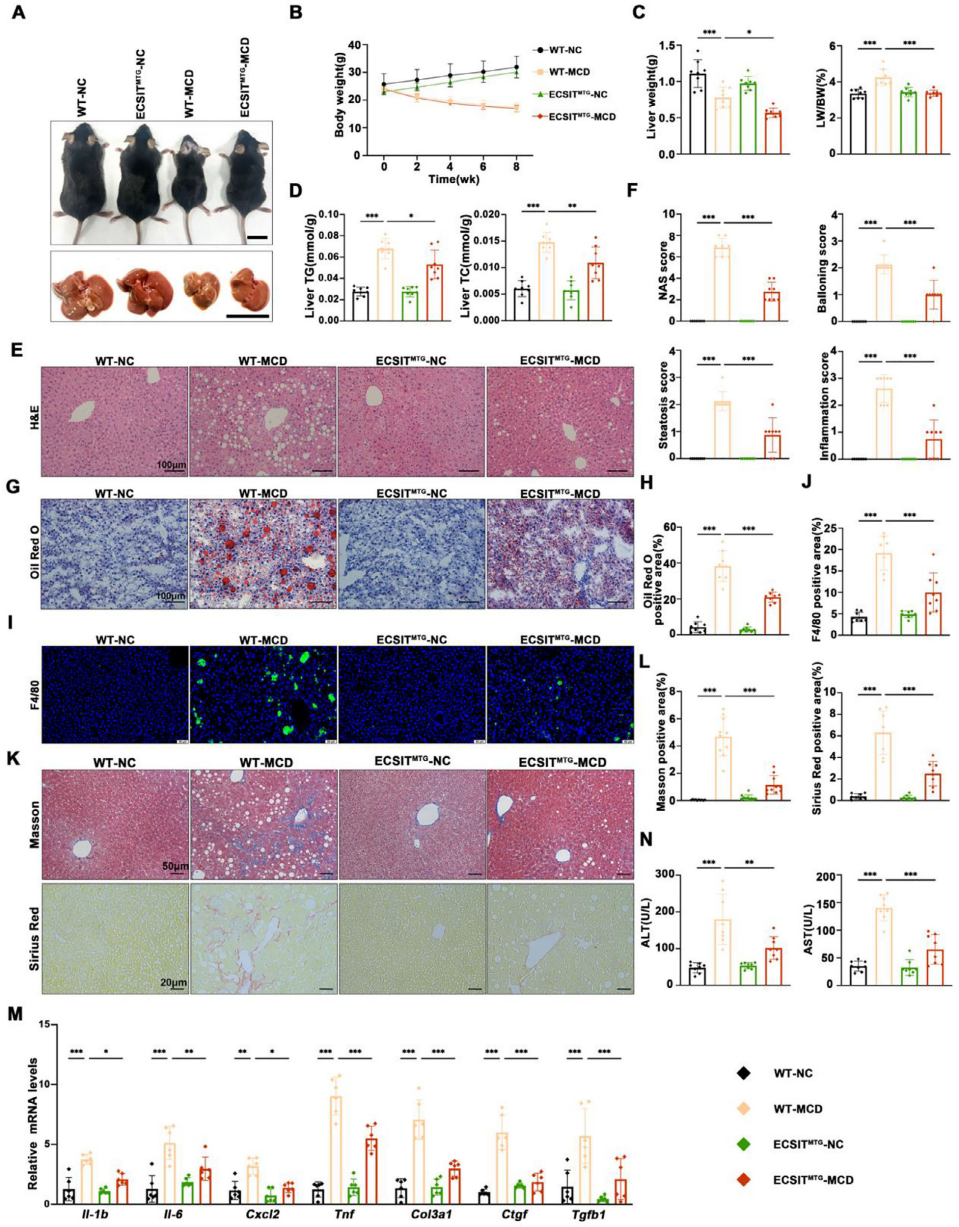
Mitochondrial ECSIT overexpression ameliorates MCD‐induced MASH in mice. WT and ECSIT^MTG^ mice were fed with NC or MCD diets for 8 weeks. A) Representative body images and liver images in the indicated groups. Scale bar: 2 cm. B,C) Records for the body weight (B), liver weight and the ratio of liver weight/body weight (%) (C) in the indicated groups. *n = 8*. D) Quantification of hepatic TG and TC levels in WT and ECSIT^MTG^ mice fed an NC or MCD diet for 8 weeks. *n = 8*. E,F) Representative pictures of H&E staining (E) and histological NAS score (F) changes in the liver sections of mice in the indicated groups. *n = 8*. Scale bar: 100 µm. G,H) Representative pictures of Oil Red O staining (G) and statistical analysis (H) showing the lipid accumulation in the liver sections of mice in the indicated groups. *n = 8*. Scale bar: 100 µm. I,J) Representative pictures of immunofluorescence staining (I) and statistical analysis (J) of F4/80 (green) in the liver sections of mice in the indicated groups (Nuclei, blue). *n = 8*. Scale bar: 50 µm. K) Representative pictures of Masson staining (upper) and Sirius Red staining (lower) indicated liver fibrosis changes in the liver sections of mice in the indicated groups. *n = 8*. Scale bar: 50 µm (upper) and 20 µm (lower). L) Statistical analysis of Masson positive area (right) and Sirius Red positive area (left). M) qPCR was performed to analyze mRNA expression of proinflammation‐related genes (*Tnf*, *Il‐6*, *Il‐1b*, and *Cxcl2*) and fibrosis‐related genes (*Col3a1*, *Ctgf*, and *Tgfb1*). *n = 6*. N) Serum ALT and AST concentrations of mice in the indicated groups. *n = 8*. All data were shown as the mean ± SD. Statistical analyses were performed by two‐way ANOVA followed by Tukey's tests for body weight and one‐way ANOVA followed by Tukey's tests for multiple comparisons. ^*^ *P *< 0.05, ^**^ *P *< 0.01, ^***^ *P *< 0.001.

Considering the heterogeneity in MASH, we conducted a deeper investigation into the function of mitochondrial ECSIT using an MCD model of MASH. In this model, MCD can induce a more serious fibrosis compared to HFHC. In alignment with the findings observed in the HFHC model, MCD diet‐fed ECSIT^MTG^ mice exhibited no significant increase in body weight compared to WT mice. However, these mice demonstrated a reduction in liver weight gain and a lower liver weight‐to‐body weight ratio following MCD feeding (Figure 4A–C). ECSIT^MTG^ mice on a MCD diet exhibited markedly attenuated hepatic lipid deposition as evidenced by significant reductions in hepatic TG and TC content (Figure 4D), and confirmed by H&E staining (Figure 4E), NAS score (Figure 4F), and Oil Red O staining (Figure 4G,H). The inflammation (Figure 4I,J), collagen deposition (Figure 4K,L), and related gene expression (Figure 4M) also showed more beneficial changes in the ECSIT^MTG^ mice compared to WT mice following MCD diet challenge. In addition, the severity of liver injury induced by MCD, as indicated by serum levels of ALT and AST, was significantly lower in ECSIT^MTG^ mice, in comparison to WT mice (Figure 4N). Finally, these results indicate that targeted mitochondrial ECSIT overexpression can alleviate MASH by inhibiting hepatic steatosis, inflammation, and fibrosis.

### 2.4 Mitochondrial ECSIT Overexpression Attenuates mtDNA Oxidative Damage and Restores Mitochondrial Integrity in MASH

**FIGURE 5:**
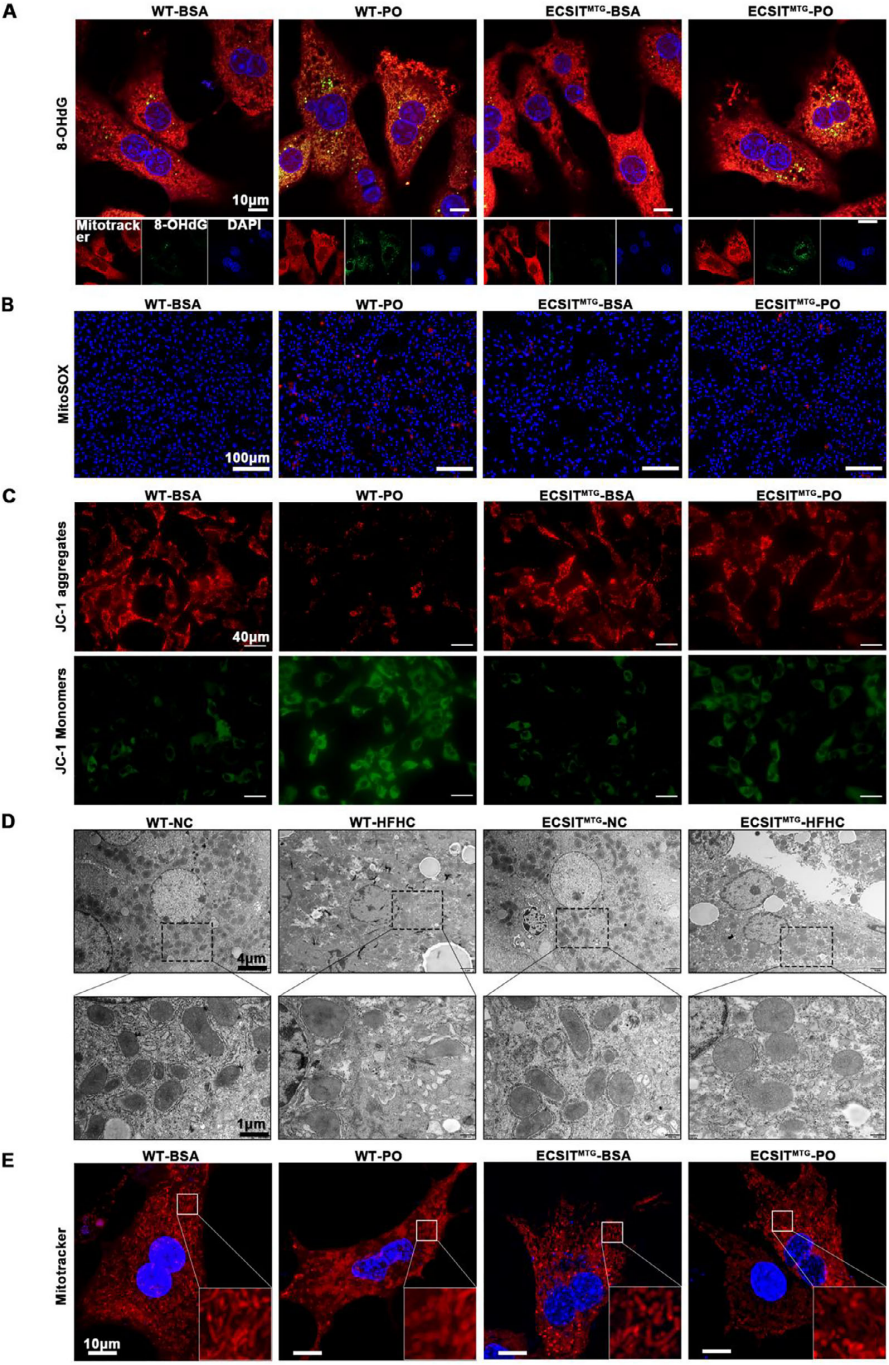
Mitochondrial homeostasis is preserved by ECSIT overexpression in MASH. Primary hepatocytes isolated from WT and ECSIT^MTG^ mice were treated with BSA or PO for 24 h. A) Representative pictures of immunofluorescence staining of 8‐OHdG (green) and Mitotracker (red) in the primary mouse hepatocytes in the indicated groups. *n = 3*. Scale bar: 10 µm. B) Representative pictures of immunofluorescence staining of MitoSOX (red) in the primary mouse hepatocytes in the indicated groups. (Nuclei, blue). *n = 3*. Scale bar: 100 µm. C) Representative pictures of immunofluorescence staining of JC‐1 in the primary mouse hepatocytes in the indicated groups. JC‐1 aggregates show red, and JC‐1 monomers show green. *n = 3*. Scale bar: 40 µm. D) Representative electron micrographs in the liver sections of WT and ECSIT^MTG^ mice following with NC or HFHC diets howing mitochondrial morphology and structure. *n = 6*. Scale bars: 4 µm (upper) and 1 µm (lower). E) Representative pictures of immunofluorescence staining of Mitotracker (red) in the primary mouse hepatocytes from WT or ECSIT^MTG^ mice treated with BSA or PO for 24 h. (Nuclei, blue). *n = 3*. Scale bar: 10 µm.

Given the critical role of ECSIT in mitochondrial function, we assessed mitochondrial alterations in the MASH model. Ox‐mtDNA damage in MASH pathogenesis was specifically analyzed via 8‐OHdG staining. PO stimulation exhibited a significant elevation in the ox‐mtDNA level, indicative of severe mtDNA oxidation (Figure 5A). Notably, mitochondrial ECSIT overexpression effectively attenuated this mtDNA damage. Subsequent mechanistic analysis showed that ECSIT suppressed mROS overproduction (Figure 5B), a key driver of mtDNA oxidation [22]. Oxidized mtDNA leaks into the cytoplasm and activates innate immune pathways such as cGAS‐STING, likely contributing to hepatic inflammation in MASH [23]. Since mitochondrial membrane potential (ΔΨm) is a key indicator of functional integrity and is linked to permeability transition, we asked whether mitochondrial ECSIT influences ΔΨm using JC‐1 fluorescence. We found that overexpression of mitochondrial ECSIT effectively prevented the PO‐induced loss of ΔΨm (Figure 5C). Concomitantly, ECSIT alleviated ultrastructural damage, including mitochondrial swelling, cristae fragmentation (Figure 5D), and network disassembly (Figure 5E), suggesting that ECSIT could maintain mitochondrial integrity. Collectively, these data demonstrate that mitochondrial ECSIT overexpression preserves bioenergetic competence (attenuating mROS, stabilizing ΔΨm, and reducing ox‐mtDNA) and mitochondrial ultrastructure (cristae integrity and network morphology), and that ECSIT mediates mitochondrial functional homeostasis to protect against hepatocyte lipotoxicity in MASH.

### 2.5 ECSIT Inhibits the Degradation of SIRT3 in a Ubiquitination‐Dependent Way

**FIGURE 6:**
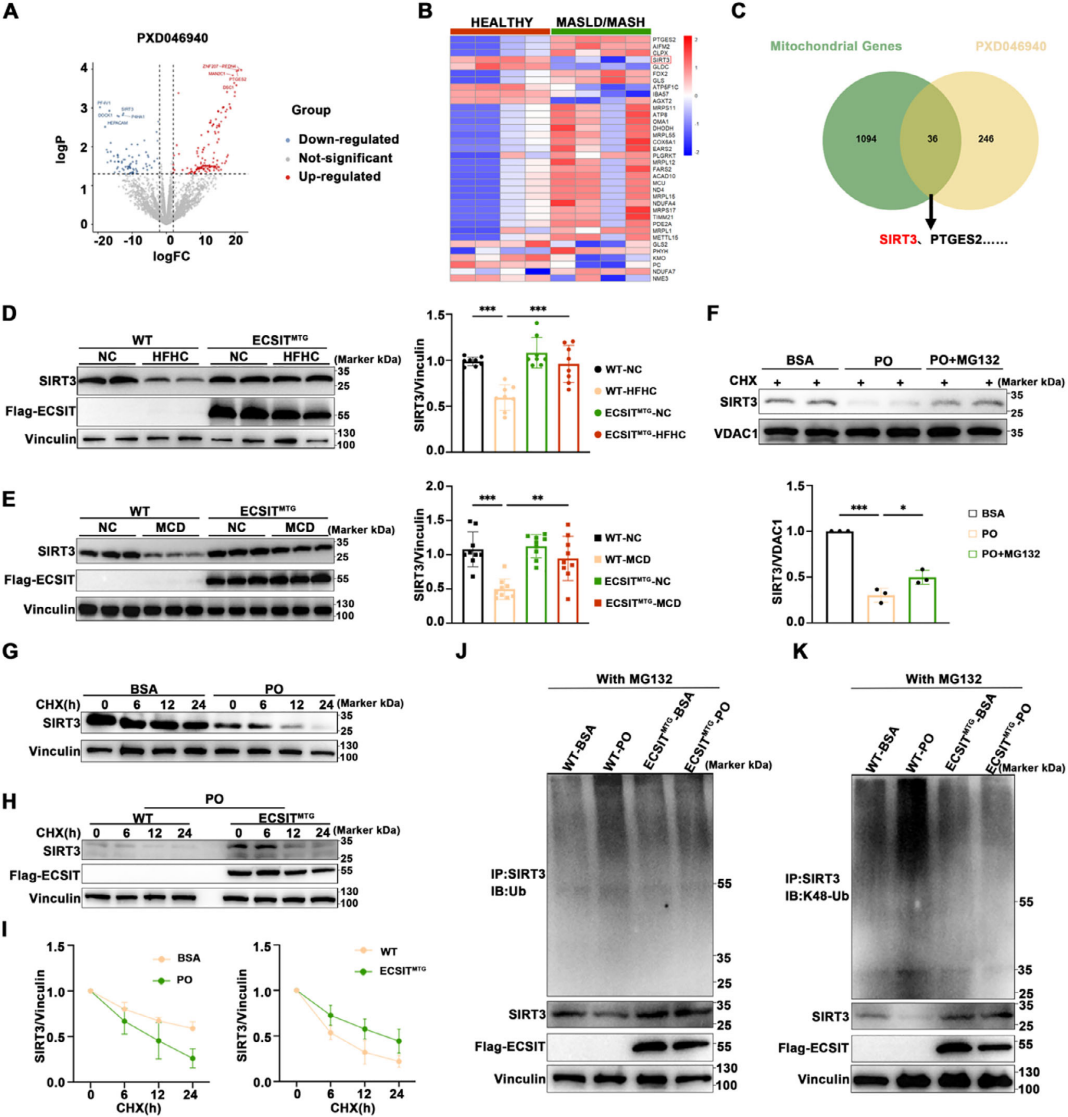
Mitochondrial ECSIT inhibits SIRT3 degradation through catalyzing its K48‐linked ubiquitination. A) Volcano plot showing differential proteins from the livers of MASLD/MASH patients compared to healthy controls in the proteomics data of PX046940. *n = 4*. B) Heatmap of differentially expressed mitochondrial proteins from proteomics data of PDX046940 between MASLD/MASH group and healthy group. *n = 4*. C) Integration of the differentially expressed proteins from proteomics analysis (PDX046940) with human mitochondrial genes. D) Representative Western blotting of SIRT3 in the liver of WT and ECSIT^MTG^ mice treated with NC or HFHC diets. *n = 8*. E) Representative Western blotting of SIRT3 in the liver of WT and ECSIT^MTG^ mice treated with NC or MCD diets. *n = 9*. F) Representative Western blotting of SIRT3 in the primary mouse hepatocytes under BSA, PO, and PO with MG132 stimulation. *n = 3*. G) Representative Western blotting of SIRT3 in the primary mouse hepatocytes were stimulated with BSA or PO and treated with Cycloheximide (CHX) for 0–24 h. *n = 3*. H) Representative Western blotting of SIRT3 in the primary mouse hepatocytes from WT or ECSIT^MTG^ mice were stimulated with PO and treated with Cycloheximide for 0–24 h. *n = 3*. I) Statistical analysis of G (left), H (right). J, K) Co‐IP results show total ubiquitination (J) and K48‐linked ubiquitination (K) of SIRT3 in the primary mouse hepatocytes from WT or ECSIT^MTG^ mice stimulated with BSA or PO after MG132 treatment. All data were shown as the mean ± SD. Statistical analyses were performed by student's t‐test for comparison between the two groups and one‐way ANOVA followed by Tukey's tests for multiple comparisons. ^*^ *P* < 0.05, ^**^ *P *< 0.01, ^***^ *P *< 0.001.

We further identified the downstream effectors of ECSIT in ameliorating MASH. We performed a 4 × 4 matrix analysis using the clinical MASH proteomics database (PDX046940). The resulting volcano plots revealed differentially expressed proteins (Figure 6A). Subsequently, a cross‐enrichment analysis identified 36 target proteins, notably SIRT3 (Figure 6B,C). Considering its crucial role in mitochondrial function, SIRT3 was analyzed in our experimental models. HFHC feeding significantly reduced hepatic SIRT3 protein levels, while mitochondrial ECSIT overexpression partially reversed this reduction (Figure 6D). Similar results were also observed in the MCD model (Figure 6E). The mechanisms responsible for both effects were further explored. MG132 partially mitigated the PO‐induced reduction in SIRT3 protein level, following protein synthesis inhibition by Cycloheximidem (Figure 6F). Furthermore, PO significantly enhanced SIRT3 protein degradation, an effect that was also attenuated by the overexpression of mitochondrial ECSIT (Figure 6G–I). These results suggest a potential role of the ubiquitin‐proteasome system in regulating SIRT3 expression during the pathogenesis of MASH. Thereafter, we examined SIRT3 ubiquitination, observing that overexpression of mitochondrial ECSIT effectively inhibited the ubiquitination of SIRT3, particularly its K48‐linked, induced by PO (Figure 6J,K). In summary, ECSIT reduces SIRT3 degradation in a ubiquitin‐dependent manner.

### 2.6 ECSIT Recruits OTUD3 to the Mitochondria and Drives K48 deubiquitination to Stabilize SIRT3

**FIGURE 7:**
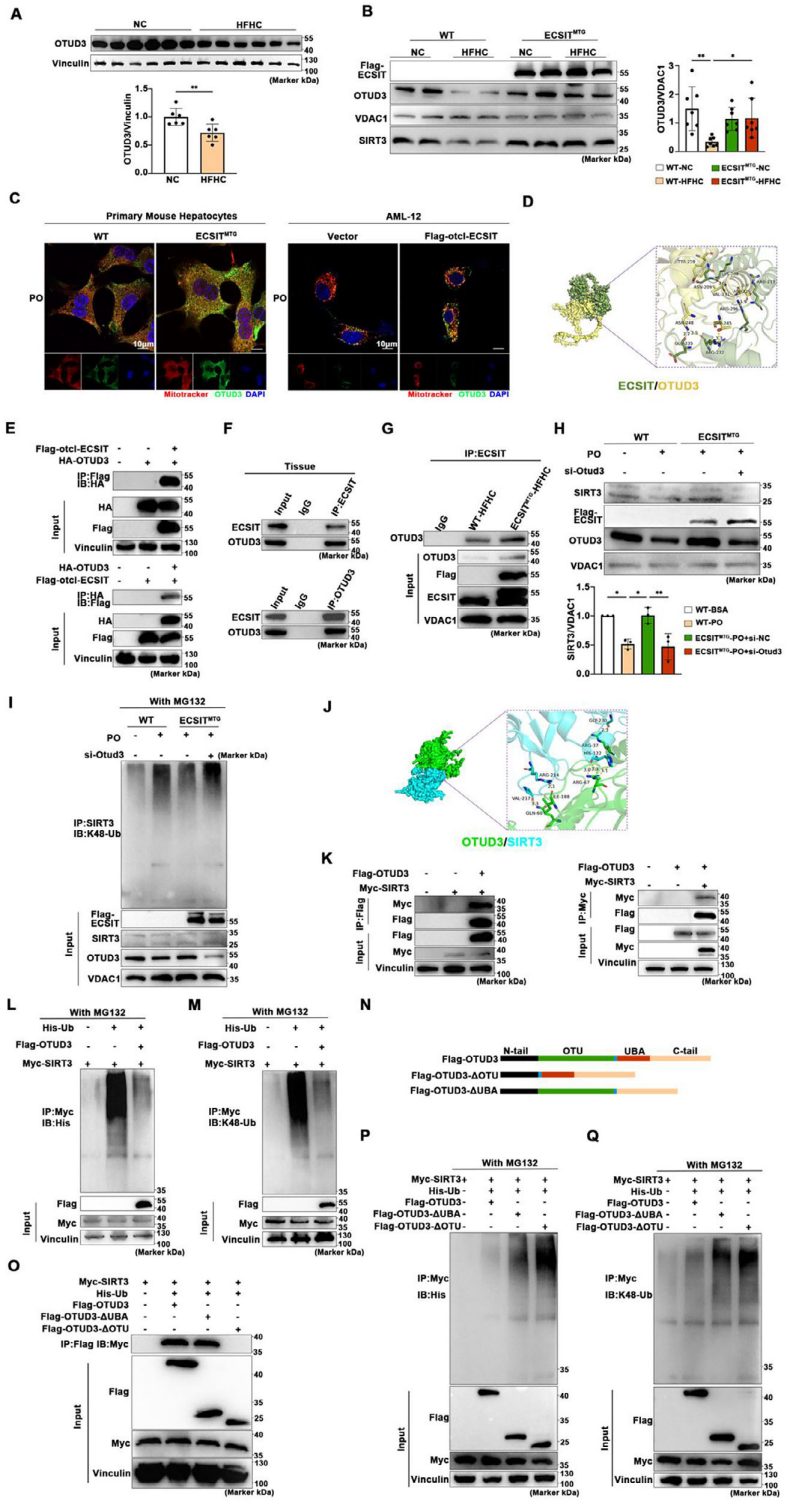
Mitochondrial ECSIT recruits OTUD3 to maintain SIRT3 protein stability. A) Representative Western blotting of total OTUD3 in the liver of WT and ECSIT^MTG^ mice treated with NC or HFHC diets. *n = 6*. B) Representative Western blotting of SIRT3 and OTUD3 in mitochondria of liver from WT and ECSIT^MTG^ mice treated with NC or HFHC diets. *n = 7*. C) Representative immunofluorescence images of mitochondria (red) and OTUD3 (green) in primary mouse hepatocytes isolated from WT and ECSIT^MTG^ mice treated PO for 24 h. *n = 4*. D) Protein‐protein docking of ECSIT (green) and OTUD3 (yellow). E) Representative Co‐IP blots showed the interaction between Flag‐otcl‐ECSIT and HA‐OTUD3 in HEK293 cells. *n = 4*. F) Representative Co‐IP blots showed the interaction between ECSIT and OTUD3 in liver samples. *n = 4*. G) Interaction between mitochondrial ECSIT and OTUD3 in the liver of WT or ECSIT^MTG^ mice under treatment of HFHC diet. *n = 4*. H) Representative Western blotting and analysis of SIRT3 in the primary mouse hepatocytes from WT and ECSIT^MTG^ mice transfected with or without si‐Otud3 and then stimulated with BSA or PO for 24 h. *n = 3*. I) Representative Co‐IP blots showed K48‐linked ubiquitination of SIRT3 in the primary mouse hepatocytes from WT or ECSIT^MTG^ mice transfected with or without si‐Otud3 and then stimulated with BSA or PO for 24 h. *n = 3*. J) Protein‐protein docking of OTUD3 (green) and SIRT3 (blue). K) Representative Co‐IP blots showed the interaction between Flag‐OTUD3 and Myc‐SIRT3 in HEK293 cells. *n = 3*. L, M) Representative Co‐IP blots showed the ubiquitination (L) and K48‐linked ubiquitination (M) of SIRT3 in HEK293 cells transfected with His‐Ub, Flag‐OTUD3 and Myc‐SIRT3. *n = 3*. N) Schematic representation of the constructed Flag‐tagged OTUD3 truncation mutants. O) Representative Co‐IP blots showed the interaction between various Flag‐tagged OTUD3 truncation mutants and Myc‐SIRT3. *n = 4*. P, Q) Representative Co‐IP blots showed the ubiquitination (P) and K48‐linked ubiquitination (Q) of SIRT3 in HEK293T cells transfected with His‐Ub, Flag‐OTUD3, Flag‐OTUD3‐ΔUBA, Flag‐OTUD3‐ΔOTU, and Myc‐SIRT3. *n = 4*. All data were shown as the mean ± SD. Statistical analyses were performed by one‐way ANOVA followed by Tukey's tests for multiple comparisons. ^*^ *P* < 0.05, ^**^ *P* < 0.01.

Next, we investigated the mechanisms by which mitochondrial ECSIT maintains the stability of SIRT3. Recent studies have employed Psort Wolf software (https://wolfpsort.hgc.jp/) to identify potential deubiquitinates localized in the mitochondria, represented by OTUD3 [16]. Western blotting analysis further confirmed the presence of OTUD3 in liver mitochondria (Figure S3A). Notably, an HFHC diet significantly reduced both the total and mitochondrial protein levels of OTUD3 in the liver, compared to the control group (Figure 7A,B). However, the overexpression of mitochondrial ECSIT mitigated this decline (Figure 7B). This effect was corroborated in vitro: mitochondrial‐targeted ECSIT overexpression in primary hepatocytes or AML‐12 cells challenged with PO increased mitochondrial OTUD3 expression (Figure 7C). Furthermore, molecular docking predicted a high‐affinity interaction between OTUD3 and ECSIT (Figure 7D), and co‐immunoprecipitation assays in HEK293T cells confirmed a physical interaction between OTUD3 and ECSIT (Figure 7E). Moreover, we discovered endogenous interactions between OTUD3 and ECSIT within tissue (Figure 7F). Under HFHC conditions, overexpression of mitochondrial ECSIT significantly enhanced their reciprocal binding (Figure 7G). Collectively, these findings suggest a model whereby ECSIT, under MASH conditions, recruits OTUD3 to the mitochondria.

Additionally, we observed that OTUD3 knockdown abolished ECSIT‐induced increase of SIRT3 protein levels under PO stimulation (Figure 7H). Correspondingly, the ubiquitination level of SIRT3 exhibited a similar profile (Figure 7I). These findings suggest that in the context of MASH, the mitochondrial ECSIT overexpression may increase SIRT3 protein levels by enhancing the mitochondrial localization of OTUD3.

Subsequently, molecular docking analysis revealed a high‐affinity interaction between OTUD3 and SIRT3 (Figure 7J). Furthermore, we confirmed that OTUD3 interacted with SIRT3 in HEK293T cells, and that OTUD3 could directly or indirectly deubiquitinate SIRT3 (Figure 7K–M). To identify the SIRT3‐binding domain of OTUD3, we constructed a series of truncation mutants (Flag‐OTUD3‐ΔUBA, Flag‐OTUD3‐ΔOTU) (Figure 7N). Deleting the OTU domain abolished the OTUD3‐SIRT3 interaction and, consequently, its deubiquitination of SIRT3 (Figure 7O). Notably, loss of the UBA domain partially impaired this deubiquitination activity (Figure 7P,Q). In summary, ECSIT stabilizes SIRT3 and blocks its K48‐linked ubiquitination by upregulating OTUD3 in the mitochondria.

### 2.7 The ECSIT‐OTUD3‐SIRT3 Signaling Axis Regulates the Progression of MASH in Response to PO In Vitro

**FIGURE 8:**
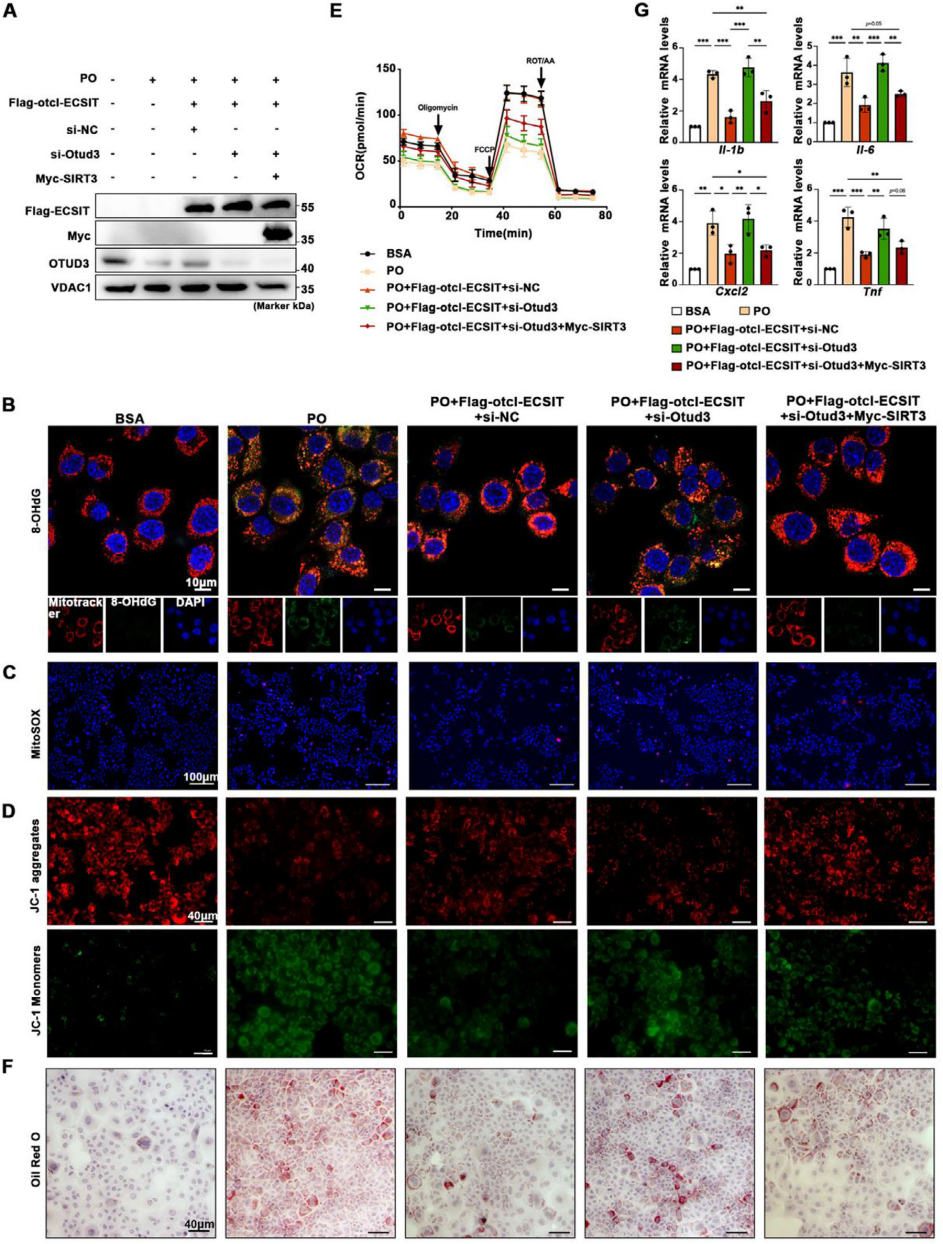
The ECSIT‐OTUD3‐SIRT3 axis tunes the progression of MASH in response to PO in vitro. Hepatocytes transfected with Flag‐otcl‐ECSIT, Myc‐SIRT3, si‐Otud3, and si‐NC according to specific groups treated with BSA or PO for 24 h. A) Representative Western blotting of Flag‐ECSIT, Myc‐SIRT3, and OTUD3 in the indicated groups. *n = 3*. B) Representative pictures of immunofluorescence staining of 8‐OHdG (green) and Mitotracker (red) in the indicated groups. *n = 3*. Scale bar: 10 µm. C) Representative pictures of immunofluorescence staining of MitoSOX (red) in the indicated groups. (Nuclei, blue). *n = 3*. Scale bar: 100 µm. D) Representative pictures of immunofluorescence staining of JC‐1 in cells in the indicated groups. JC‐1 aggregates showed red, and JC‐1 monomers showed green. *n = 3*. Scale bar: 40 µm. E) Oxygen consumption rate (OCR) in the indicated groups. *n = 5*. F) Representative pictures of Oil Red O staining showing the lipid accumulation in cells in the indicated groups. Scale bar: 40 µm. *n = 4*. G) qPCR was performed to analyze mRNA expression of proinflammation‐related genes (*Tnf*, *Il‐6*, *Il‐1b*, and *Cxcl2*) in the indicated groups. *n = 3*. All data were shown as the mean ± SD. Statistical analyses were performed by one‐way ANOVA followed by Tukey's tests for multiple comparisons. ^*^ *P* < 0.05, ^**^ *P* < 0.01, ^***^ *P* < 0.001.

To delineate the role of the mitochondrial ECSIT‐OTUD3‐SIRT3 signaling axis in MASH, we transfected hepatocytes with plasmids or siRNA targeting these molecules prior to PO stimulation (Figure 8A). Knockdown of OTUD3 abolished the protective effect of mitochondrial ECSIT overexpression against PO‐induced mitochondrial dysfunction, as evidenced by increased ox‐mtDNA (8‐OHdG staining, Figure 8B) and mROS (MitoSOX Red staining, Figure 8C), as well as decreased ΔΨm (JC‐1 staining, Figure 8D). These changes were partially reversed by SIRT3 overexpression. ECSIT is required for the assembly of mitochondrial complex I (CI) and the expression of its components. Loss of ECSIT function impairs oxidative phosphorylation (OXPHOS) [21]. To directly assess whether the ECSIT‐OTUD3‐SIRT3 axis regulates mitochondrial respiratory function, we measured the oxygen consumption rate (OCR) in cells under PO‑induced lipotoxic stress. Compared to control cells exposed to PO stress alone, cells overexpressing mitochondrial‐targeted ECSIT exhibited a pronounced enhancement in mitochondrial respiration. This was evidenced by significant increases in key OCR parameters, including basal respiration, maximal respiratory capacity. Strikingly, the robust increase in OCR mediated by ECSIT overexpression was substantially attenuated upon knockdown of OTUD3. This result positions OTUD3 as a crucial downstream mediator of ECSIT's pro‐respiratory effects. Furthermore, reintroducing SIRT3 expression in OTUD3‐deficient cells effectively rescued the OCR deficit (Figure 8E; Figure S4A,B). This rescue experiment demonstrates that SIRT3 functions as a key effector within this axis, acting downstream of OTUD3 to ultimately enhance OXPHOS. Furthermore, Oil Red O staining revealed that ECSIT overexpression attenuated PO‐induced lipid accumulation, whereas OTUD3 knockdown negated this effect, which was subsequently rescued by SIRT3 overexpression (Figure 8F). A parallel regulatory pattern was observed in key inflammatory genes at the mRNA level (Figure 8G). Collectively, mitochondrial ECSIT employs OTUD3 to stabilize SIRT3, thus mitigating PO‐induced lipotoxicity, inflammation, and mtDNA oxidation in hepatocytes.

## 3 Discussion

**FIGURE 9:**
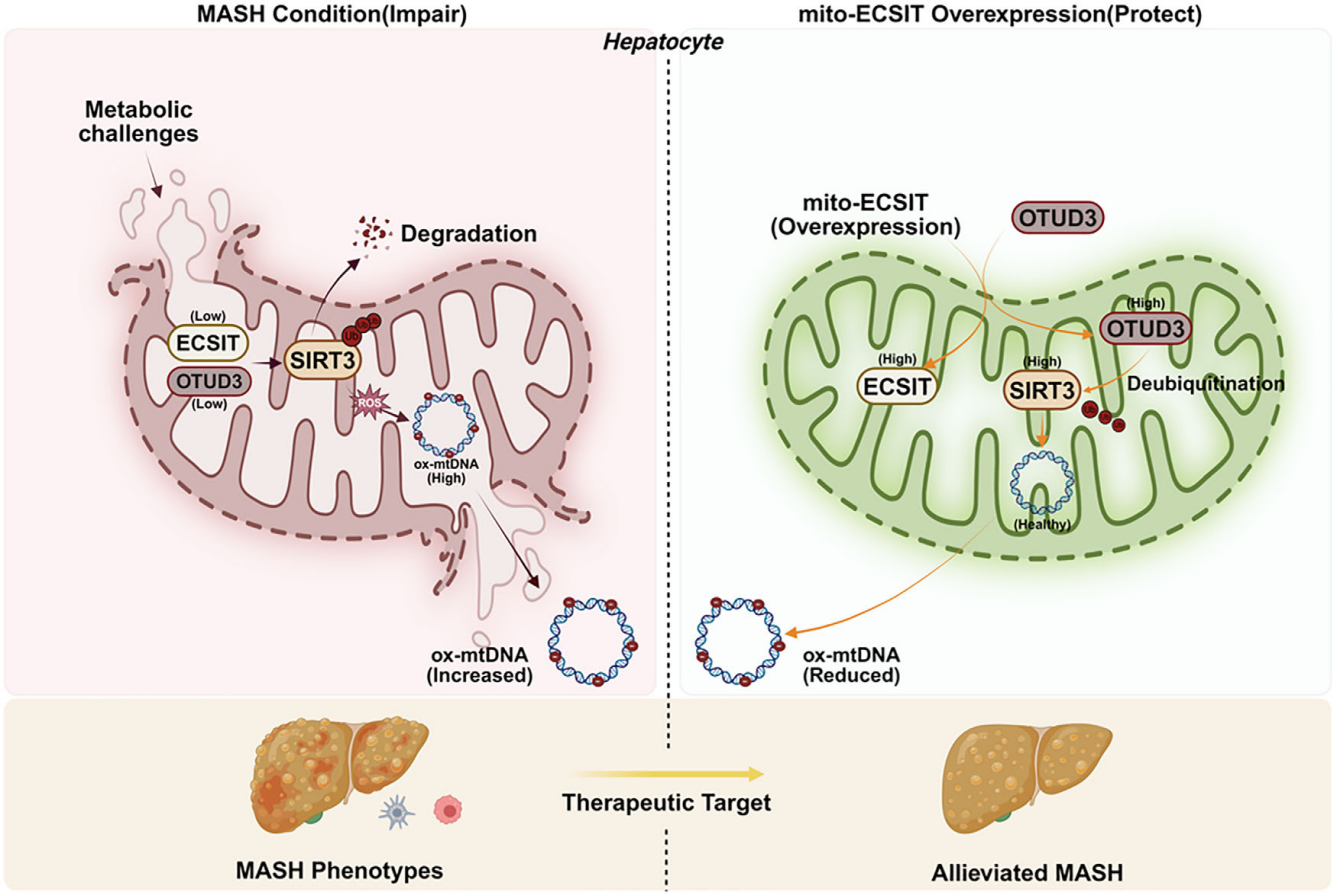
Schematic representation of the ECSIT/OTUD3/SIRT3 axis in MASH. Mitochondrial ECSIT expression is reduced during MASH pathogenesis. Overexpression of mitochondrial ECSIT mitigates steatohepatitis and maintains hepatic metabolic homeostasis. Mechanistically, ECSIT enhances intramitochondrial OTUD3 expression to stabilize SIRT3 through deubiquitination, thus preserving mitochondrial function and impeding disease progression.

The link between MASH and mitochondrial dysfunction may inspire the design for new efficacious therapeutic strategies. Mitochondrial pathways are often disrupted during the pathogenesis of MASH [24]. Our study revealed downregulation of key mitochondrial proteins, including ECSIT, SIRT3, and OTUD3, during MASH progression. Overexpression of mitochondrial ECSIT concomitantly upregulated intramitochondrial OTUD3 and SIRT3 to attenuate steatohepatitis. Moreover, OTUD3 deubiquitinated to stabilize SIRT3 at the protein level, thereby inhibiting mtDNA oxidation and reducing lipotoxicity‐induced metabolic derangements to counteract MASH (Figure 9).

MtDNA is a 16.5‐kilobase pair double‐stranded circular molecule inherently vulnerable to oxidative damage [25]. Ox‐mtDNA can escape the mitochondrial matrix to activate the NLRP3 inflammasome [26]. Specifically, mtDNA damaged in MASH can be released from hepatocytes to act as a damage‐associated molecular pattern. This extracellular mtDNA engages Toll‐like receptor 9 on target cells, thereby amplifying the inflammatory response and worsening steatohepatitis [27]. Established as a key regulator of mtDNA integrity, SIRT3 has demonstrated its protective effects against mtDNA damage in the lungs (e.g., protecting alveolar epithelial cells) and heart (e.g., mitigating doxorubicin‐induced cardiotoxicity via 8‐oxoguanine DNA glycosylase 1 maintenance). Inspiringly, our study revealed that overexpression of mitochondrial ECSIT reduced ox‐mtDNA levels in MASH by upregulating SIRT3. While the role of SIRT3 silencing in MASH pathogenesis is established, the upstream mechanisms that regulate its expression remain poorly understood [28, 29]. Ubiquitination and deubiquitination cooperate to decide the stability and function of mitochondrial proteins in diverse pathologies. For instance, USP14 mildens doxorubicin‐induced cardiotoxicity by deubiquitinating and stabilizing SIRT3, and USP11 delays intervertebral disc degeneration via deubiquitinating and stabilizing SIRT3 [30, 31]. Building on these findings, we demonstrate a novel pathway through which mitochondrial ECSIT uplifts the mitochondrial expression of deubiquitinase OTUD3 to enhance the K48‐linked ubiquitination of SIRT3. The observed improvements in mitochondrial membrane potential and ROS levels likely contribute to reduced inflammatory activation. This ameliorated cellular stress could, in turn, lessen the propensity for hepatocyte apoptosis, highlighting a potential dual protective role of the ECSIT‐OTUD3‐SIRT3 axis in MASH.

Deubiquitination is closely engaged in the pathophysiology of MASH [32]. Multiple deubiquitinating enzymes can modulate MASH progression through key metabolic and stress‐response pathways involving, apoptosis signal‐regulating kinase 1 [33], mechanistic target of rapamycin [34], TGF‐β‐activated kinase 1 [35], and notably, the Sirtuins family [36]. Unprecedently, our present study demonstrated that deubiquitinase OTUD3 is expressed in the mitochondria. OTUD3 has been characterized as a broad‐spectrum deubiquitinase capable of cleaving diverse ubiquitin linkages, including K48 chains; meanwhile, its role in stabilizing metabolic regulators has been increasingly recognized, as exemplified by its regulation on peroxisome proliferator‐activated receptor δ stability [15]. Here, we identify SIRT3 as a novel substrate of the deubiquitinase OTUD3. OTUD3 stabilizes SIRT3 through deubiquitination, thereby maintaining mitochondrial function. Molecular docking and domain deletion assays further reveal a specific interaction between the OTU domain of OTUD3 and SIRT3. Intriguingly, we found that not only the OTU domain but also the UBA domain of OTUD3 is required for its full deubiquitinating activity toward SIRT3.

ECSIT, a multifunctional adaptor protein, is implicated in diverse pathological processes, ranging from neurodegeneration to oncogenesis [17, 37, 38, 39, 40]. Recent studies have recognized it as a player bridging oxidative stress, inflammatory signaling, and mitochondrial homeostasis [41]. Initially identified as a cytosolic adaptor in Toll‐like receptor (TLR) signaling, ECSIT activates NF‐κB during innate immune responses. However, an N‐terminal MTS (amino acids 1–48) directs a distinct pool of ECSIT to the mitochondria. Our laboratory research has demonstrated that mitochondrial ECSIT can appeal mitochondrial STAT3 to prevent pressure overload‐induced cardiac hypertrophy [20]. Here, the involvement of mitochondrial ECSIT in metabolic liver diseases was clarified. Our results indicate that down‐regulation of mitochondrial ECSIT expression represents an early event in the transition beyond simple steatosis. In the slow‐progression model induced by an HFHC diet, a significant decrease in ECSIT was observed only after 12 weeks, which is a time point that typically coincides with the progression from isolated MASL to full MASH, characterized by overt inflammation and hepatocellular injury. Similarly, in the rapidly advancing MCD diet model, ECSIT expression exhibited only a declining trend at 3 weeks, with a pronounced downregulation becoming apparent only when the disease had fully evolved into overt MASH by 8 weeks. This in vivo timeline was further supported by in vitro experiments, in which significant ECSIT downregulation in primary hepatocytes was induced only under conditions of high‐concentration or prolonged lipotoxic stress, such as those that recapitulate the sustained metabolic insult characteristic of the MASH stage. Collectively, these findings suggest that mitochondrial ECSIT functions primarily as a MASH‐stage regulatory factor. Its downregulation appears to be closely associated with the inflammatory phase of metabolic liver disease rather than with the initial establishment of steatosis. We therefore propose that altered ECSIT expression or activity may contribute critically to the pathogenic shift from benign lipid accumulation to steatohepatitis, rather than playing a central role in the onset of simple steatosis. Critically, mitochondrial ECSIT overexpression mitigated oxidative injury to relieve core disease manifestations, such as steatosis, inflammation, and fibrosis, suggesting its potential as a therapeutic target.

While our study provides substantial evidence supporting a critical role for mitochondrial ECSIT in MASH pathogenesis, we must also consider the well‐documented function of ECSIT as an adaptor in the cytosolic TLR signaling pathway [21, 42]. Given the prominence of inflammation in MASH, it is plausible that the cytosolic pool of ECSIT could contribute to the observed inflammatory phenotype. In our model, the primary insult appears to be mitochondrial dysfunction evidenced by increased ROS production, impaired mitochondrial membrane potential, and increased ox‐mtDNA. We therefore speculate that the mitochondrial role of ECSIT is the dominant driver in our experimental context, potentially creating a pro‐inflammatory milieu that secondarily amplifies other signaling pathways, including possibly the cytosolic TLR pathway. This proposes a compelling model of inter‐compartmental crosstalk: mitochondrial ECSIT deficiency establishes a foundation of metabolic distress, which may then lower the threshold for activation of cytosolic inflammatory cascades. While our data demonstrate that rescuing mitochondrial ECSIT function is sufficient to ameliorate both metabolic and inflammatory hallmarks of MASH, future studies employing cytosolic‐only ECSIT construct are warranted to definitively dissect the individual contributions of its mitochondrial and cytosolic pools to the complex pathophysiology of MASH.

Insulin resistance (IR) is recognized as a major causal factor in hepatic metabolic disorders [43]. Notably, amelioration of hepatic fat accumulation is known to improve whole‐body insulin sensitivity significantly, even in the absence of substantial weight loss [44]. For instance, in a clinical trial of Chiglitazar‐a drug used to treat hypertriglyceridemia and IR‐patients with MASLD showed significantly reduced liver fat content and improved markers of liver injury, yet their body weight did not differ significantly from the placebo group [45]. Here, in HFHC‐induced MASH mouse model, we observed that amelioration of hepatic lipid metabolism and IR was not accompanied by weight reduction. Interestingly, MCD model presents a unique scenario where steatosis and fibrosis occur alongside significant weight loss. Within this model, drugs that improve hepatic lipid metabolism and fibrosis generally act against this characteristic weight loss [46]. In our study, overexpression of mitochondrial ECSIT in the MCD model led to improved liver parameters. A concomitant tendency toward weight rebound was observed, but it did not reach statistical significance, potentially due to the limited sample size. Notably, metabolically healthy obesity is associated with lower ectopic fat but preserved or even expanded subcutaneous adipose tissue capacity, which may explain the dissociation between improved insulin sensitivity and unchanged body weight in our model [44]. While whole‐body overexpression model does not exclude contributions from other tissues, our findings establish a central role for hepatocytes. Investigating liver‐organ crosstalk and tissue‐specific ECSIT signaling will be an important focus of future studies.

Despite our in vitro findings that delineate a mechanistic link between ECSIT and SIRT3, our study acknowledges a limitation in the form of absent in vivo data specifically interrogating this axis. A definitive in vivo validation, for instance through rescuing SIRT3 in a mitochondrial ECSIT‐deficient setting, would solidify our conclusion. Therefore, while our cell‐based data robustly demonstrate that SIRT3 is both necessary and sufficient for ECSIT‐mediated benefits, we propose that the direct in vivo validation of this interaction constitutes a critical and compelling direction for future research.

## 4 Conclusion

The discovery of mitochondrial ECSIT opens new avenues for targeted intervention in MASH. By promoting the mitochondrial expression of OTUD3 to maintain the stability of the key deacetylase SIRT3, it effectively clears oxidized mtDNA and halts disease advancement. This mechanism unveils novel insights into MASH pathology and may hold promise as a potential therapeutic strategy.

## 5 Experimental Section

### 5.1 Animals and Mouse Models of MASH

Male 8‐week‐old C57BL/6J mice (20–25g) were obtained from the Animal Core Facility of Nanjing Medical University (Nanjing, China). The targeting vector pCAG‐otcl‐ECSIT‐3 × Flag‐BPA (construction scheme is detailed below) was linearized and purified by restriction enzyme and absolute ethanol, respectively, and then transferred into embryonic stem cells. The mitochondrial ECSIT overexpression mice were constructed by microinjection according to the standardized method. The Animal Care and Use Committee of Nanjing Medical University approved all animal research, which was conducted in accordance with the National Research Council's Guide for the Care and Use of Laboratory Animals (Permit Number: IACUC‐2412060). To establish the MASH model induced by MCD (Methionine and Choline Deficient L‐Amino Acid), 10‐week‐old male mice were fed with an MCD (Jiangsu Synergy Pharmaceutical Bioengineering Co., China) diet for 8 weeks. To establish the MASH model induced by HFHC (protein, 14%; fat, 42%; carbohydrate, 44%; cholesterol, 0.2%), 10‐week‐old male mice were fed with an HFHC (Jiangsu Synergy Pharmaceutical Bioengineering Co., China) diet for 16 weeks.

### 5.2 Mouse Primary Hepatocytes Isolation and Treatment

Hepatocytes were isolated from 8 to 10‐week‐old male WT and ECSIT^MTG^ mice using standard protocols [47]. The mice were anesthetized and perfused with liver perfusion medium and liver digestion medium through the inferior vena cava. Subsequently, the liver was dissected, minced, and filtered through a 70 µm cell strainer. The suspension was centrifuged at 500 rpm for 5 min at 4°C. The supernatant collected was centrifuged at 300–400g to obtain non‐parenchymal cell precipitates. The initial pellet, enriched with hepatocytes, was collected and further purified by Percoll density gradient centrifugation. The freshly prepared MPHs were then inoculated into 6‐cm cell culture dishes at a density of 1 × 10^6^ cells per dish and supplemented with William's Medium E (Gibco, Waltham, MA, USA) containing 10% fetal bovine serum (FBS, Gibco, Waltham, MA, USA) and 1% penicillin‐streptomycin (Beyotime, Shanghai, China) at 37°C in 5% CO_2_. The culture medium was replaced after 6 h of cellular adhesion. To inhibit the proteasomal degradation, the cells were treated with 25 mM MG132 (Selleck, State of Texas, USA) for 4 h. Cycloheximide (CHX; 50 µg/mL, Selleck, State of Texas, USA) was used in the CHX chase assay.

### 5.3 Cell Culture

HEK 293T cell line (RRID:CVCL_0063) and AML‐12 cell line(RRID:CVCL_0140) were provided by the Cell Bank of the Chinese Academy of Sciences, culturing in Dulbecco's Modified Eagle Medium (DMEM, Invitrogen Corporation, USA) supplemented with 10% FBS, 1% penicillin, and streptomycin at 37°C in 5% CO_2_. The authenticity of the cell lines was verified. Both cell lines were tested free of contamination. These cells were maintained under these conditions for the specified experiments.

### 5.4 In Vitro Lipotoxic Model

Cells were exposed to 0.3 mm palmitic acid (Sigma, St. Louis, USA) and 0.5 mm oleic acid (Sigma, St. Louis, USA), dissolved in 1% fatty acid‐free bovine serum albumin, to create an in vitro lipotoxic model. Hepatocytes isolated from mice and AML‐12 cells were cultured in PO‐containing medium for 24 h. Subsequently, liver cells were harvested for further analysis.

### 5.5 Isolation of Mitochondria

The lysate was centrifuged at 1000g for 10 min at 4°C to isolate cytosolic proteins in the supernatant. The remaining cell pellet was resuspended in disruption buffer and disrupted using a blunt‐ended needle and syringe. Following a second centrifugation at 1000g for 10 min at 4°C, the supernatant was transferred to a clean 1.5 mL tube for another centrifugation at 6000g for 10 min at 4°C. The resulting pellet was purified using a mitochondria‐purifying buffer and further centrifugated at 14 000g for 15 min at 4°C to obtain mitochondria.

### 5.6 RNA Extraction and qRT‐PCR

The Total RNA Extraction Reagent (Vazyme, Nanjing, China) was used to extract total RNA from cells. According to the directions provided by Takara's PrimeScriptTM RT Reagent Kit with gDNA Eraser (Perfect Real Time) Kit (TaKaRa, Beijing, China), RNA was reverse transcribed into cDNA. On the StepOnePlusTM Real‐Time PCR System with Tower (Applied Biosystems, MA, USA), the product was amplified and detected. Invitrogen created the primers for qRT‐PCR, which were utilized to assess mRNA expression. The cycle times (Ct) for each target PCR was compared to determine the relative gene expression, as previously mentioned. The primer sequences are shown in Table S1.

### 5.7 Western Blot

RIPA buffer (Beyotime, Shanghai, China) was used to harvest the cells, and a BCA Protein Assay Kit (Thermo Scientific, Waltham, MA, USA) was applied to determine the protein content. Proteins were denatured and then put through a Western blot. Then, equal quantities of indicated proteins were separated by 10% SDS‐PAGE gels and then transferred to PVDF membranes (Millipore, St. Louis, USA). Having been blocked with 5% skim milk in Tris‐buffered saline/Tween 20 (TBST) for 1.5 h at room temperature, the PVDF membranes with proteins were incubated with the indicated primary antibodies at 4°C overnight and incubated with HRP‐conjugated secondary antibodies for 1.5 h at room temperature. Signals were then visualized using an ECL Kit (Thermo, Waltham, MA, USA) in a ChemiDoc MP Imaging System (Bio‐Rad, Hercules, CA, USA). Vinculin, Tubulin, and VDAC1 was used as a loading control. The antibodies used are listed in Table S2.

### 5.8 Mitochondrial mROS, JC‐1 Staining

Cells were washed with pre‐cooled PBS and incubated in the dark with 5 µm MitoSOX (Life Technologies, Carlsbad, CA, USA) or JC‐1 staining solution from enhanced mitochondrial membrane potential assay kit with JC‐1 (Beyotime, Shanghai, China) for 15 min, followed by a 10‐min incubation with Hoechst at 37°C. Fluorescence was measured using a microscope (OLYMPUS TH4‐200, Tokyo, Japan). Images were captured from five randomly selected fields of view.

### 5.9 Glucose Tolerance Test (GTT) and Insulin Tolerance Test (ITT)

GTT and ITT were conducted after 16 weeks of HFHC feeding. After fasting for 6 h, the mice were injected with 1 g/kg glucose or 0.75 IU/kg insulin intraperitoneally, and the level of blood glucose was measured at indicated time points. Subsequently, the areas under the curve (AUC) were calculated by using the conventional trapezoid rule.

### 5.10 Histological Examinations

Liver tissue samples from mice were fixed in 4% paraformaldehyde for 48 h, embedded in paraffin, and sectioned into 7‐µm slices. H&E staining (Solarbio, Beijing, China) was conducted on paraffin‐embedded liver sections to visualize histological features. Oil Red O staining (Solarbio, Beijing, China) was conducted on OCT‐embedded liver sections to evaluate hepatic steatosis. Masson's trichrome staining (Solarbio, Beijing, China) was conducted to assess liver fibrosis. The images were observed, captured by a BX51 microscope (OLYMPUS, Tokyo, Japan) and analyzed with the ImageJ software. Sirius Red staining relies on the pathological staining service of Wuhan Servicebio Company (China).

### 5.11 Liver Triglyceride (TG) and Total Cholesterol (TC)

Levels of TG and TC in livers were analyzed using a test kit from Nanjing Jiancheng Bioengineering Institute, according to the manufacturer's protocol.

### 5.12 Oxygen Consumption Rate(OCR)

AML‐12 cells were adherent in an Agilent Seahorse XF24 Cell Culture Microplate, treated with plasmid (Flag‐otcl‐ECSIT, Myc‐SIRT3) and si‐RNA (si‐NC, si‐Otud3) for 24 h, and then treated with BSA or PO for 24 h. Later, replace with 360 µL of Substrate‐Limited Assay Media and incubate in a CO2‐free incubator for 60 min. Add Oligomycin, FCCP(Carbonyl cyanide p‐trifluoromethoxyphenylhydrazone), and AA/ROT (Antimycin/Rotenone)working solutions to the probe plate to give final well concentrations of 2, 1, and 2 µm/2 µm, respectively. Put the probe plate into the analyzer for calibration and put itinto the cell plate for detection after completion.

### 5.13 Immunofluorescence Staining

Liver tissue sections were dewaxed in xylene, rehydrated through a graded ethanol series. Liver tissue sections were sequentially washed in PBS, permeabilized with 0.1% Triton X‐100 for 15 min, blocked with 5% normal goat serum (BioGenex, Fremont, CA, USA) for 1 h at room temperature, and incubated with primary antibodies according to the manufacturer's protocols. Sections were incubated with Alexa Fluor 488‐ or 594‐conjugated secondary antibodies (1:500 dilution; Life Technologies, Carlsbad, CA, USA) at 37°C for 90 min in the dark with DAPI nuclear staining. Microscope (Zeiss, Baden‐Wurttemberg, Germany) was used to capture images from five randomly selected fields per group.

### 5.14 Co‐Immunoprecipitation

Cells were harvested and lysed following established procedures. The lysates were then mixed with specific antibodies and left to incubate at 4°C overnight. Subsequently, 30 µL of protein G agarose beads (Cytiva, Shanghai, China) were introduced and co‐incubated with the immune complexes for 4 h at 4°C. Following three rounds of washing with cold wash buffer and one with lysis buffer, the immunoprecipitates were suspended in 30 µL of lysis buffer for Western blotting to identify the target proteins.

### 5.15 Plasmid and Si‐RNA Transfection

The OTCL and ECSIT fragment was amplified from mouse cDNA by RT‐PCR, and 3 × Flag fragment was amplified from a plasmid template. Both fragments were then cloned into a NheI/NotI‐digested and dephosphorylated pCAG vector. Following transformation into DH5α competent cells, ampicillin screening and PCR identification yielded a positive clone containing the constructed plasmid pCAG‑otcl‑ECSIT‑3 × Flag. Plasmids encoding Flag‐tagged OTUD3 and Myc‐tagged SIRT3 were purchased from MIAOLING (China). The OTUD3 domain deletion mutant plasmid was constructed using the Flag‐OTUD3 plasmid as the template. Specific primers were designed to amplify the desired deletion fragment, which was then inserted into the corresponding vector via restriction digestion and ligation. The ligation product was transformed into competent *E. coli*, and positive clones were selected through antibiotic screening and confirmed by DNA sequencing. Si‐Otud3 and si‐NC were purchased from GenePharma (Shanghai, China). For transient transfection, the cells were transduced with the indicated plasmids using Lipofectamine 2000 reagent (Invitrogen) according to the manufacturer's recommendations. In brief, when the cells reached 60%–70% confluence, they were placed in Opti‐MEM and transfected with the plasmids using Lipofectamine 2000 reagent. After 6 h, the transfection mixture was replaced with DMEM‐supplemented 10% FBS.

### 5.16 Statistical Analysis

All data were presented as means ± SD. Prism 9 was used for statistics and graphics. Two‐tailed unpaired Student's t‐test compared two groups under conditions of homogeneity of variance (*p* > 0.1). For comparisons involving more than two groups, one‐way or two‐way analysis of variance (ANOVA) with post hoc Turkey's test correction was applied. Significance was set at *p *< 0.05. Figure legends provide additional statistical details, including sample size (n) for each analysis.

## Author Contributions

J.T.L., Y.H.L., Q.Z., Y.Q.J., T.T.T., and P.X.S. were involved in the conceptualization of the study. Y.Q.J., T.T.T., P.X.S., X.F.C., C.H.W., Q.Y.W., S.H.C., and L.L.Q. designed and performed the experiments. J.T.L., Y.H.L., and Q.Z. supervised the project. Y.Q.J., T.T.T., and P.X.S. analyzed the data, Y.Q.J., T.T.T., and P.X.S. prepared the figures, Y.Q.J., T.T.T., and P.X.S. edited the manuscript. All authors were involved in the review of the manuscript. J.T.L., Q.Z., Y.H.L., and Q.C. acquired funding for the studies.

## Funding

This work was supported by grants from the National Natural Science Foundation of China (81900221), the Natural Science Foundation of Shanghai City (25ZR1401391).

## Conflicts of Interest

The authors declare no conflicts of interest.

## Supporting information




**Supporting File**: advs74283‐sup‐0001‐SuppMat.docx

## Data Availability

The data that support the findings of this study are available from the corresponding author upon reasonable request.
