# Supplementary material for: Targeted Mitochondrial ECSIT Overexpression Attenuates MASH by Increasing OTUD3 Expression
Source: Adv Sci (Weinh). 2026 Feb 4;13(22):e18974. doi: 10.1002/advs.202518974 (PMC13088300; doi:10.1002/advs.202518974)
Supplement: Supplementary file 1 — Supporting File: advs74283‐sup‐0001‐SuppMat.docx [file ADVS-13-e18974-s001.docx]

**Targeted** **Mitochondrial ECSIT Overexpression Attenuates MASH by increasing OTUD3 expression**

Yuqing Jiang ^1,^ †, Tingting Tong ^1,^ †, Pengxi Shi^1,^ †, Xiaofan Chen ^1^, Chenhao Wang ^1^, Qingyuan Weng ^2^, Sihan Chen ^2^, Linli Que ^1^, Qi Chen ^1^, Yuehua Li ^1, *^, Qiang Zhu ^3, *^, Jiantao Li ^1, *^

^1^ Key Laboratory of Targeted Intervention of Cardiovascular Disease, Collaborative Innovation Center for Cardiovascular Disease Translational Medicine, School of Basic Medical Science, Nanjing Medical University, Nanjing, Jiangsu 211166, China.

^2^ The First Clinical Medical College, Nanjing Medical University, Nanjing, Jiangsu 211166, China.

^3^ Department of Organ Transplantation, Eastern Hepatobiliary Surgery Hospital, Naval Medical University, Shanghai 200433, China

†These authors contributed equally: Yuqing Jiang, Tingting Tong, Pengxi Shi

*****Corresponding authors:

Jiantao Li, Ph.D., Assoc.Prof.

Department of Pathophysiology

Nanjing Medical University

Nanjing, Jiangsu, China, 211166

E-mail: ljt@njmu.edu.cn

Qiang Zhu, MD, Ph. D.

Department of Organ Transplantation

Eastern Hepatobiliary Surgery Hospital

Naval Medical University

Shanghai, China,200438

Email: [zhu20081023@yeah.net](mailto:zhu20081023@yeah.net)

Yuehua Li, MD, Ph. D.

Professor in the Department of Pathophysiology

Nanjing Medical University

Nanjing, China, 211166

E-mail: [yhli@njmu.edu.cn](mailto:yhli@njmu.edu.cn)

**Supplemental Material**

**Table S1. Primer sequences for real-time-PCR**

| Gene | Forward | Reverse |
| --- | --- | --- |
| Mus *Il-1b* | AAATACCTGTGGCCTTGGGC | CTTGGGATCCACACTCTCCAG |
| Mus *Il-6* | CCGGAGAGGAGACTTCACAG | ACTCCAGAAGACCAGAGGAAAT |
| Mus *Cxcl2* | CTTACACCTGACTGGCACCC | CTTAGGGACACACAGCGACC |
| Mus *Tnf* | AGGGTCTGGGCCATAGAACT | CCACCACGCTCTTCTGTCTAC |
| Mus *Col3a1* | TGACTGTCCCACGTAAGCAC | GAGGGCCATAGCTGAACTGA |
| Mus *Ctgf* | CCCAACTATGATGCGAGCCA | TGACTAGGGGCAGAGGATGT |
| Mus *Tgf* | ATACAGGGCTTTCGATTCAGC | GTCCAGGCTCCAAATATATAG |
| *18S rRNA* | AGTCCCTGCCCTTTGTACACA | CGATCCGAGGGCCTCACTA |

**Table S2. Antibodies for Western Blots**

| **Name** | **Supplier** | **Cat no.** |
| --- | --- | --- |
| ECSIT Rabbit anti-Mouse Polyclonal (aa241-290) Antibody | LS-Bio | [LS-C31040](https://www.lsbio.com/antibodies/ecsit-antibody-aa241-290-wb-western-ls-c31040/29509) |
| Rabbit monoclonal anti- Ubiquitin(K48) | Abcam | ab140601 |
| Mouse monoclonal anti-Ubiquitin | [Santa Cruz Biotechnology](https://www.baidu.com/link?url=bTkVyj7t40SOfMwfk6VhEUHE4YNFEsu-A3d3v0PXhTCZBDZ83utnXQQYwGsxG8UY&wd=&eqid=a789183601169df00000000567e7b356) | sc-166553 |
| Myc Tag Mouse Monoclonal Antibody | Beyotime | AF0033 |
| HA tag Polyclonal antibody | Proteintech | 51064-2-AP |
| 6*His, His-Tag Monoclonal antibody | Proteintech | 66005-1-AP |
| Mouse monoclonal anti-FLAG-tag | Sigma-Aldrich | F1804 |
| Anti-8-Hydroxy-2'-deoxyguanosine antibody | Abcam | ab48508 |
| Vinculin Rabbit mAb | ZEN-BIOSCIENCE | R26084 |
| α-Tubulin Mouse Monoclonal Antibody | Beyotime | AF2827 |
| SIRT3 Polyclonal antibody | Proteintech | 10099-1-AP |
| OTUD3 Polyclonal antibody | Proteintech | 29622-1-AP |
| VDAC1/Porin Recombinant antibody | Proteintech | 81538-1-RR |
| F4/80 | Abcam | ab6640 |
| Mouse IgG | Beyotime | A7028 |
| Rabbit IgG | Beyotime | A7016 |

**
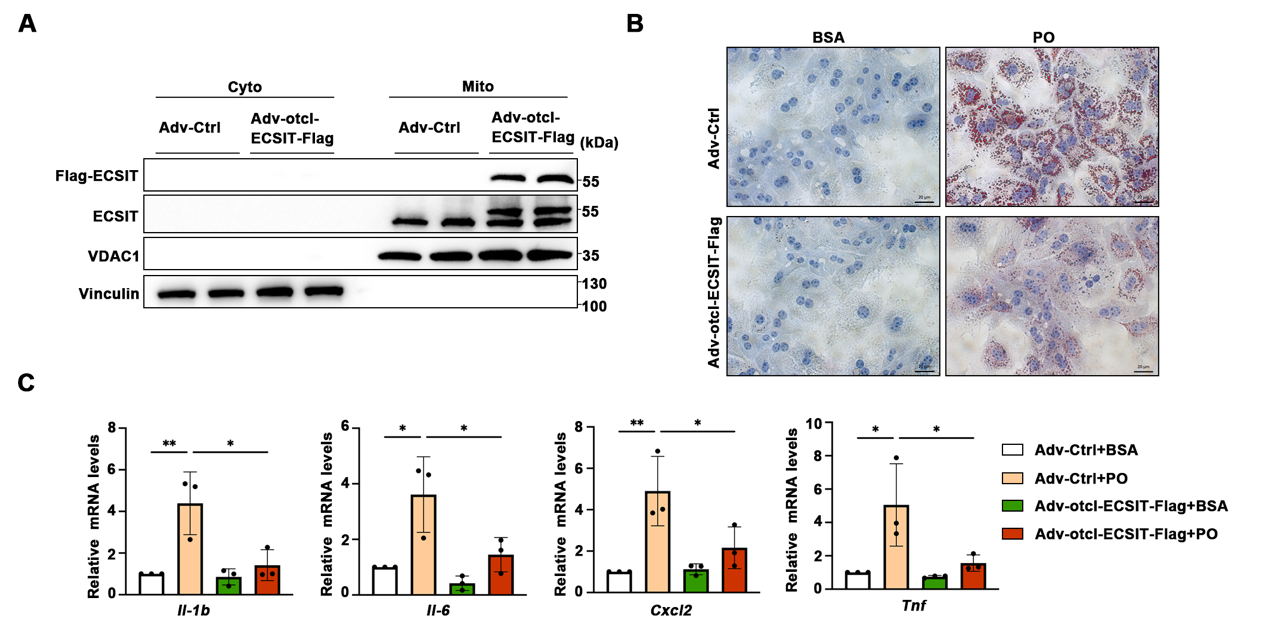
**

**Figure S1.** **Mitochondrial ECSIT shields hepatocytes against lipid buildup and inflammation induced by PO stimulation.** **A**) Primary mouse hepatocytes were infected using Adv-Flag-otcl-ECSIT or Adv-Ctrl. Representative Western blotting was used to verify the overexpression efficiency. *n=3*. **B**) Representative pictures of Oil Red O staining showing the lipid accumulation in primary mouse hepatocytes were infected using Adv-otcl-ECSIT-Flag or Adv-Ctrl with treatment of BSA or PO for 24 h in the indicated groups. Scale bar: 20 μm. *n=3*. **C**) qPCR was performed to analyze mRNA expression of proinflammation-related genes (*Tnf*, *Il-6*, *Il-1b* and *Cxcl2*) in the indicated groups. *n=3*. All data were showed as the mean ± SD. Statistical analyses were performed by one-way ANOVA followed by Tukey’s tests for multiple comparisons. **P* <0.05, ***P* <0.01.

**
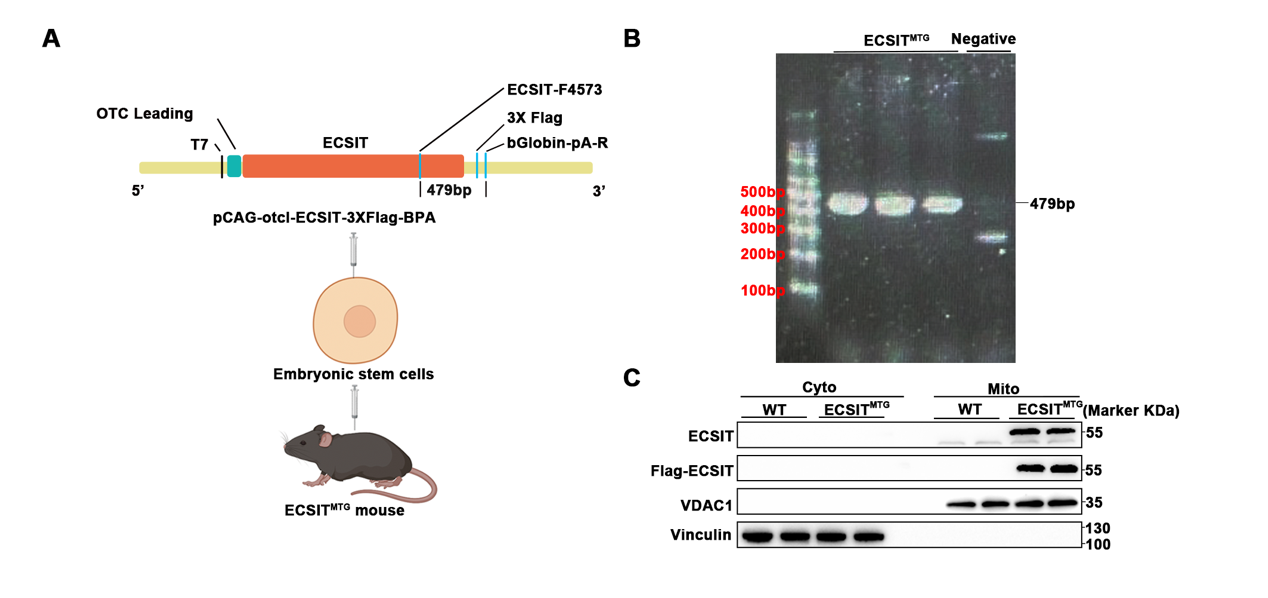
**

**Figure S2.** **Construction and validation of mitochondria-targeted ECSIT transgenic mice. A**) Schematic representation of the construction of mitochondrially targeted ECSIT overexpressing mice. **B**) Identification of overexpression efficiency of mitochondria targeted ECSIT. **C**) Representative Western blotting of identification of overexpression efficiency of mitochondria targeted ECSIT.

**
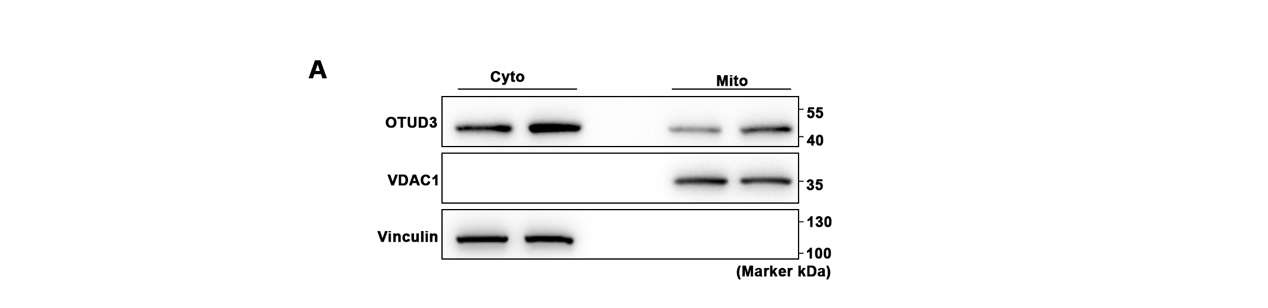
**

**Figure S3.** **Validation of mitochondrial localization of OTUD3 in the liver. A**) Representative Western blotting of OTUD3 in the liver of mice.


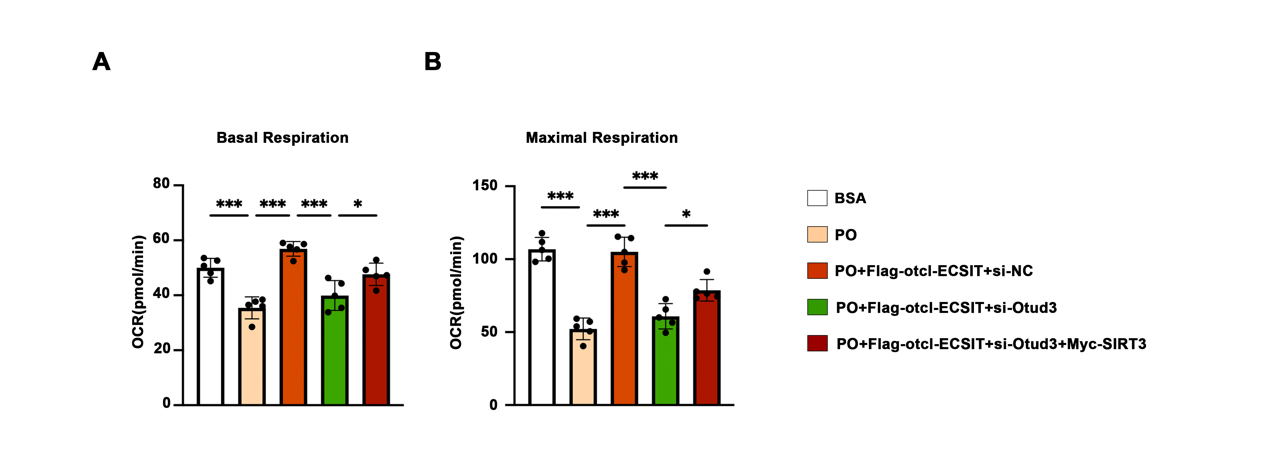


**Figure S4. ECSIT‑OTUD3‑SIRT3 axis enhances mitochondrial basal and respiration under PO stimulation. A**, **B**) Hepatocytes transfected with Flag-otcl-ECSIT, Myc-SIRT3, si-Otud3 and si-NC according to specific groups treated with BSA or PO. Quantification of basal respiration (**A**) and maximal respiration (**B**) in the indicated groups. All data were showed as the mean ± SD. Statistical analyses were performed by one-way ANOVA followed by Tukey’s tests for multiple comparisons. *n=5*. **P* <0.05, ****P* <0.001.
